# MiRNA-1/133a Clusters Regulate Adrenergic Control of Cardiac Repolarization

**DOI:** 10.1371/journal.pone.0113449

**Published:** 2014-11-21

**Authors:** Johannes Besser, Daniela Malan, Katharina Wystub, Angela Bachmann, Astrid Wietelmann, Philipp Sasse, Bernd K. Fleischmann, Thomas Braun, Thomas Boettger

**Affiliations:** 1 Department of Cardiac Development and Remodeling, Max-Planck-Institute for Heart and Lung Research, Bad Nauheim, Germany; 2 Institut für Physiologie I, Life & Brain Center, Universität Bonn, Bonn, Germany; The University of Tennessee Health Science Center, United States of America

## Abstract

The electrical properties of the heart are primarily determined by the activity of ion channels and the activity of these molecules is permanently modulated and adjusted to the physiological needs by adrenergic signaling. miRNAs are known to control the expression of many proteins and to fulfill distinct functions in the mammalian heart, though the *in vivo* effects of miRNAs on the electrical activity of the heart are poorly characterized. The miRNAs miR-1 and miR-133a are the most abundant miRNAs of the heart and are expressed from two miR-1/133a genomic clusters. Genetic modulation of miR-1/133a cluster expression without concomitant severe disturbance of general cardiomyocyte physiology revealed that these miRNA clusters govern cardiac muscle repolarization. Reduction of miR-1/133a dosage induced a longQT phenotype in mice especially at low heart rates. Longer action potentials in cardiomyocytes are caused by modulation of the impact of β-adrenergic signaling on the activity of the depolarizing L-type calcium channel. Pharmacological intervention to attenuate β-adrenergic signaling or L-type calcium channel activity *in vivo* abrogated the longQT phenotype that is caused by modulation of miR-1/133a activity. Thus, we identify the miR-1/133a miRNA clusters to be important to prevent a longQT-phenotype in the mammalian heart.

## Introduction

To maintain appropriate blood flow the vertebrate heart needs to fulfill highly regulated and coordinated contraction of atria and ventricles. This process is controlled by the propagation of the electrical excitation throughout the electrical syncytium of the heart. The spreading of the excitation as well as the contractile response of the cardiomyocytes is modulated by the autonomous nervous system allowing both the adjustment of heart rate and strength of contraction. Adrenergic signaling plays a key part in the autonomous regulation and with its many downstream effectors it needs to be coordinated and highly regulated to allow the differentiated response of the heart to the needs of the physiology and to ensure the function of the heart. miRNAs control the expression of proteins at the posttranscriptional level and are therefore part of the regulatory options to control the function of such signaling networks [1]. The miRNAs miR-1 and miR-133a are the most abundant miRNAs found in the heart and these miRNAs are encoded in two clusters in the genome. The miR-1-1/133a-2 cluster on mouse chromosome 2 and the miR-1-2/133a-1 cluster on mouse chromosome 18 give rise to identical mature miR-1 or miR-133a molecules, respectively. Possible functions of the miRNAs have been addressed by different studies. For miR-1-2 a role in the regulation of cardiac conduction has been described using miR-1-2 deficient mice [2]. This model revealed that miR-1-2 might regulate protein abundance of IRX5, which represses transcription of KCND2 [3], encoding the potassium channel subunit Kv4.2 that encodes the cardiac transient outward potassium current I_to,f_ [4]. This finding has been correlated to the ECG abnormalities, namely changes in the morphology and length of the R-wave of the ECG [2]. In addition, loss of miR-1-2 caused ventricular septal defects (VSDs) with partial penetrance and this has been attributed to dysregulation of gene programs influenced by direct miR-1 targets like Hand2 [2]. Interestingly, in the recently described miR-1-1 single mutant mice an obvious phenotype with neonatal lethality, VSDs and cardiac fibrosis was only detected in a pure 129 inbreed background [5]. Here changes in several ECG-parameters were described and upregulation of Irx5 mRNA was observed, but it remains unclear, whether this causes molecular changes that might affect the electrical properties of the heart [5]. Deletion of miR-133a from the one or other genomic cluster did not cause an apparent phenotype [6]. However, complete deletion of both miR-133a copies from the genome reduced viability of newborn animals, led to increased proliferation of neonatal cardiomyocytes and ectopic expression of smooth muscle genes, an effect described to be mediated by the direct miR-133a target SRF [6]. Previously we have shown that the clustered miRNAs miR-1 and miR-133a act as functional units, with miR-1 negatively regulating the abundance of myocardin that in turn enhances the expression of the miR-1/133a clusters by direct transcriptional activation [7]. Thus the miR-1/133a clusters and myocardin constitute a feedback-loop and myocardin activates transcription of smooth muscle-related genes, amongst others the potassium channel Kcnmb1 that on the other hand is repressed by miR-133a. Thus these clustered miRNAs may cooperate to regulate different molecules in common biological processes. Loss of both miR-1/133a clusters is lethal at an embryonic stage earlier than E12.5 proving the function of miR-1/133a clusters in regulatory processes that are fundamental for embryonic cardiomyocyte specification. We demonstrated that this phenotype is caused to a large extend by the loss of the regulatory interaction between miR-1 and myocardin [7]. However, whereas deletion of both miR-1/133a clusters was embryonic lethal, we did not observe a major phenotype if only a single miR-1/133a cluster was deleted from the genome. On the other hand, adenoviral overexpression [8] or increased expression of miR-1 or miR-133a in rabbit cardiomyocytes during chronic heart failure [9] have been linked to modulation of intracellular calcium release and promotion of arrhythmogenesis due to dysregulation of phosphatase activities. Thus, besides the fundamental role of miR-1/133a in development the potential relevance of the most abundant miRNAs in the heart in regulating electrophysiological properties of cardiomyocytes is still unclear to date.

We show here, that the two miR-1/133a expressing genomic clusters are involved in regulation of the impact of adrenergic signaling in cardiomyocytes and in the modulation of the electrical properties of the adult heart. Deletion of one or the other miR-1/133a cluster specifically led to longer QT intervals in the ECG. This finding is underscored at the single cell level by prolonged action potential duration and this appears to be related to altered L-type calcium-currents in cardiomyocytes. The observed longQT as well as the altered calcium-current are triggered by adrenergic signaling, indicating that the miR-1/133a clusters are essential for the maintenance of signaling pathways involved in adjustment of cardiomyocyte repolarization.

## Methods

### Ethics statement

All animal experiments were in accordance with German animal protection laws and were approved by the local governmental animal protection committee (Regierungspraesidium Darmstadt - Hessen, Germany; B2/272, B2/199).

### Mouse models

Deletion of the miR-1/133a clusters located on mouse chromosome 2 and 18 has been described previously [7]. To further exclude potential effects of the neomycin resistance cassette used to delete the miR-1-2/133a-1 genomic region on the expression of the Mib1 gene, that contains the miR-1-2/133a-1 locus in an intron, we deleted the selection cassette using loxP sites flanking the neoR cassette. A CMV-Cre mouse [10] was used to delete the selection cassette in heterozygous miR-1-2/133a-1 mutant mice. Cre was detected by PCR (TAAACTGGTCGAGCGATGGATTTCC, CATATCTCGCGCGGCTCCGACACGG). The WT and the mutant allele after deletion of the selection cassette (miR-1-2/133a-1^del^) were detected by PCR with a mutant specific (GCTAACATTTCTGAATACACTTAAGACTCTG), a WT specific (AACACGTGAATTTTCTGTTTAACAA) and a common primer (CATAAAACACTGGCTGTCCATGTGT), resulting in a 420 bp (mutant) and a 230 bp (WT) PCR product. All experiments were performed in a mixed 129/C57 background.

### Histology and Immunostainings

Muscle tissues were dissected and snap frozen in Propane/Isopentane (1∶2) on liquid nitrogen. Adult hearts were fixed in 4% PFA/PBS and then incubated in 30% Sucrose/PBS over night at 4°C. Tissues were embedded in Tissue Tek and cryosectioned. Sections were fixed in 4% PFA/PBS, washed 3 times with PBS and stained with DAPI and fluorophore-labeled *Triticum vulgaris* lectin (Sigma; L5266 or L4895; 1∶200). A Z1 Axioimager (Zeiss) and ImageJ v1.45 was used to determine the area of muscle fibers and cardiomyocytes.

Muscle sections were fixed with 4% PFA/PBS and treated with 0.02% pepsin solution (Sigma, P6887) in 0.2 N HCl for 2 min and blocked for 45 min (2% BSA, 3% NGS, 0.5% NP-40). Anti-Myosin antibodies (Sigma, M8421, anti-skeletal-slow; 1∶900 or M4276, anti-skeletal-fast; 1∶450) were incubated over night at 4°C. Biotinylated universal antibody (Vector, BA-1400) 1∶1000 was applied for 2 h at room temperature. DAB-staining using the VECTASTAIN Elite ABC Kit was used to visualize fast and slow muscle fibers.

### Isolation of cardiomyocytes and non-cardiomyocytes from heart

Isolation of adult mouse cardiac myocytes was done as described previously [11]. In brief, the isolated heart was cannulated via the aorta and rinsed with calcium-free perfusion buffer to remove erythrocytes. The heart was enzymatically digested by perfusion with digestion buffer until it became swollen and turned slightly pale. Atria and outflow tract were removed and the ventricle was dissociated in stop buffer. Isolated myocytes were washed and the Ca^2+^ content was adjusted up to 1 mM stepwise. Cells were centrifuged twice for 1 min at 300 rpm. The cell pellet constituting the cardiomyocyte fraction was taken up in culture medium and seeded on Laminin coated dishes. Culture medium was changed after 2–3 hrs. The supernatant obtained from the centrifugation steps contains the non-cardiomyocyte fraction. Non-cardiomyocytes were collected by centrifugation for 2 min at 2000 rpm, these cells were grown on uncoated dishes in DMEM (1 g glucose, 10% FCS, 1% PSG) for 3–5 days. The culture medium of cardiomyocytes was changed after 12–16 hrs and cells were cultured overnight. For activation of the β-adrenergic signaling cardiomyocytes were washed once with cell rinse buffer (0.15 M NaCl, 40 mM Tris, 1 mM EDTA, pH 7.4) 18 h after isolation and stimulated with or w/o 1 µM Isoproterenol (I5627, Sigma) for 5 min at RT. Subsequently, supernatant was removed and SDS containing buffer added followed by immediate harvesting and sonication.

### Northern blots

Total RNA from different tissues of adult mice was isolated using the Trizol method (Invitrogen). 5 µg of RNA were separated in a 15% denaturing polyacrylamide TBE-Urea gels (Invitrogen) and blotted to a Hybond-XL membrane (Amersham) that was subsequently hybridized with (γ-32P)ATP labeled miR-1 (ATACATACTTCTTTACATTCCA) or miR-133a (CAGCTGGTTGAAGGGGACCAAA) and U6 snRNA (ATATGGAACGCTTCACGAATT) probe diluted in ULTRAhyb buffer (Ambion) at 30°C overnight. The membrane was washed with SSC/SDS containing buffer and signals were detected using imaging plates scanned with a BAS-2500 reader (Fujifilm) and analyzed with AIDA software (v4).

### Affymetrix analysis

Total RNA was isolated from hearts sampled at daytime (mean heart rate at daytime 480±14 bpm) from approx. 18 weeks old male mice utilizing the Trizol method (Invitrogen). RNA quality was verified using Agilent Bioanalyser and the RNA 6000 Nano Kit. RNA was labeled following the protocol of Affymetrix. Labeled samples were hybridized to Affymetrix GeneChip Mouse 430 2.0 arrays, processed, scanned and analyzed (RMA with Affymetrix Expression console, statistical analysis using students t-test with DNAStar Arraystar 5). Microarray data are deposited at http://www.ebi.ac.uk/arrayexpress (Acc#E-MTAB-2727).

### Quantitative RT-PCR

Total RNA was isolated from tissue or cells using Invitrogen Trizol protocol. Purified RNA was reverse transcribed applying Invitrogen SuperScript II Reverse Transcriptase (18064-014) protocol or Applied Biosystems TaqMan MicroRNA Reverse Transcription Kit (4366596). Applied Biosystems TaqMan assays were performed following the manufacturer's instructions using a StepOnePlus Real-Time PCR System: Acta2, Mm01204962_gH; Tgln, Mm00441661_g1; Kcne1, Mm01215533_m1; Kcnd2, Mm01241698_g1; Gja1, Mm01179639_s1; Myocd, Mm01325105_m1; hsa-miR-1, 002222; hsa-miR-133a, 002246; U6 snRNA, 001973.

### Western blot

Tissues or cell culture samples were homogenized and gathered in SDS containing extraction buffer, followed by sonication. Concentration was determined using the Bio-Rad DC Protein Assay for colorimetric protein concentration measurement. NuPAGE Novex 4–12% Bis-Tris Gels (Invitrogen) were loaded with 7.5–20 µg of protein. Denaturating Western blots were performed using Invitrogen NuPAGE electrophoresis protocol and solutions. Proteins were transferred to Nitrocellulose membrane and stained with Red Alert. Membranes were blocked by incubation with 5% Skim Milk Powder (70166, Fluka) in TBST for 1 h at RT. Primary antibodies were diluted in 3% BSA or 3% milk powder in TBST and incubated over night at 4°C. Following antibodies and dilutions were used: mouse α-B56alpha (1∶1000; 610615, BD), rabbit α-Cacna1c (1∶200; ab58552, abcam), rabbit α-Gapdh (1∶1000; 2118, Cell Signaling Techn.), rabbit α-Histone H3 (1∶1000; 9715, Cell Signaling Techn.), rabbit α-Irx5 (1∶1000; ARP37245 P050, Aviva Systems Biology), goat α-Kcne1 (1∶500; SC-16796, Santa Cruz), mouse α-Myocd (1∶500; MAB4028, R&D), rabbit α-pCamKII Thr286 (1∶1000; 3361, Cell Signaling Techn.), goat α-Plb Ser16 (1∶50; sc-12963, Santa Cruz), rabbit α-Plb Thr17 (1∶50; sc-17024-R, Santa Cruz), rabbit α-pPKA C Thr197 (1∶1000; 5661, Cell Signaling Techn.), rabbit α-pTroponin I Ser23/24 (1∶1000; 4004, Cell Signaling Techn.), rabbit α-RyR2 Ser2808 (1∶5000; A010-30, Badrilla), rabbit α-RyR2 Ser2814 (1∶5000; A010-31, Badrilla), rabbit α-Sorcin (1∶500; PA5-28359, Pierce), rabbit α-SRF (1∶500; SC-335, Santa Cruz). HRP coupled secondary antibodies (goat-α-mouse, 1858413, Pierce or goat-α-rabbit, 1858415, Pierce or rabbit-α-goat, A5420 Sigma) were diluted 1∶5000 in 3% Skim Milk Powder/TBST or 5% BSA/TBST and incubated for 1 h at RT. Signal detection was performed using chemiluminescence (Femto-Kit, Pierce) and a VersaDoc system (Biorad) with the software Quantity One. Signal intensity was quantified with Quantity One or ImageJ v1.45 h.

### ECG

Surface ECG was measured using a custom made amplifier with a suitable tube to immobilize mice and electrodes positioned to the location of the paws of the mice. Amplified signals were digitized, recorded and analyzed using Powerlab and LabChart7 software (AdInstruments). For ECG analysis mouse preset settings were used, with an average time of 30 s and a maximum RT interval of 140 ms. To manipulate heart rates, mice were anesthetized after initial measurement by application of 2–2.5% Isoflurane/O_2_ (20 ccm/min) to the mice. For normalization PR, QRS, QT and ST durations at different heart rates were calculated from slopes (ΔQT/ΔRR). In selected experiments 5 mg/kg Propranolol (Sigma, P0884) was applied i.p. 10 min before ECG recording. Verapamil (Sigma, V4629) was administered via drinking water at a concentration of 1 g/liter [12]. Adult mice were treated for 4 weeks before measurement. Alternatively ECG of mice was recorded using TA10EA-F20 implantable telemetric transmitters (Data Sciences International). The electrodes of the transmitter were placed subcutaneously left and right on the thorax. Data were recorded every 30 min for 300 s for at least 6 days. First recordings were done 7 days after implantation of transmitter. Data were sampled with Dataquest A.R.T. 4.0 with a sample rate of 500 Hz and with a filter cut-off of 100 Hz. ECG parameters at different heart rates were obtained by different activity of the mice during day- and nighttime.

### MRI measurements

Cardiac MRI measurements were performed on a 7.0 T Bruker Pharmascan, equipped with a 300 mT/m gradient system, using a custom-built circularly polarized birdcage resonator and the IntraGateTM self-gating tool [13]. The parameters for identification of the ECG were adapted for one heart slice and transferred afterwards to the navigator signals of the remaining slices. Thus the in-phase reconstruction of all pictures is guaranteed. MRI data were analyzed using Qmass digital imaging software (Medis). Mice were measured under volatile Isoflurane (1.5–2.0%) anesthesia. The measurement is based on the gradient echo method (repetition time  = 6.2 ms; echo time  = 6.0 ms; field of view  = 2.20×2.20 cm; slice thickness  = 1.0 mm; matrix  = 128×128; repetitions  = 100). The imaging plane was localized using scout images showing the 2- and 4-chamber view of the heart, followed by acquisition in short axis view, orthogonal on the septum in both scouts. Multiple contiguous short-axis slices consisting of 7 to 10 slices were acquired for complete coverage of the left and right ventricle.

### Electrophysiological recordings

Ventricular cardiomyocytes were isolated from 30 to 36 week-old control or miR-1-1/133a-2, miR-1-2/133a-1 mice, as previously described [14]. Briefly, hearts were perfused in the Langendorff mode with Tyrode solution (135 mM NaCl, 4 mM KCl, 1 mM MgCl_2_, 2.5 mM HEPES, 5 mM glucose, 25 mM butanedione monoxime; pH 7.4) for 5 min at 37°C and then with Tyrode containing 50 µM CaCl_2_, 0.8 mg/ml collagenase B (Roche) and 0.3 mg/ml trypsin (Invitrogen) for 12–13 min. The ventricles were cut in small pieces and mechanically dissociated, then cells were filtered through a nylon mesh and the pellet was resuspended in Tyrode containing 50 µM CaCl_2_ and 5% FCS. [Ca^2+^] in the buffer was increased in four steps from 50 µM to 1.8 mM over 40 min.

Single cells were plated at low density on laminin-coated (0.1%) coverslips in normal external solution. Patch-clamp experiments were performed using an EPC10 amplifier (Heka) in the whole cell configuration. I_Na_ was recorded in the voltage clamp mode, for recording of peak I_Na_ and recovery from inactivation of I_Na_ the pipette solution contained (in mM) 3 NaCl, 133 CsCl_2_, 2 MgCl_2_, 2 NaATP, 2 TEACl, 10 EGTA and 5 HEPES (pH 7.3, CsOH), the external solution: 7 NaCl, 133 CsCl_2_, 1.8 CaCl_2_, 1.2 MgCl_2_, 5 Hepes, 11 glucose, 0.005 nifedipine (pH 7.4, CsOH). For the recovery of inactivation kinetics peak I_Na_ was determined in response to pairs of depolarizing voltage steps from −100 mV to 10 mV with increasing delays between the two pulses (from 1.5 ms to 57 ms); for quantitation, I_Na_ amplitude of the second pulse was normalized to the first pulse, plotted against the delay and the data fitted with a mono-exponential decay. Peak I_Na_ densities were measured from the first 40 ms lasting pulse from a holding potential of −100 mV to −10 mV of the recovery from inactivation protocol. For measuring peak I_Ca,L_, cardiomyocytes were held at a holding potential of −80 mV, then 50 ms long depolarizing voltage steps to −40 mV were applied to inactivate I_Na_, followed by 300 ms depolarizing voltage steps to +10 mV at a frequency of 0.2 Hz. For recording of IVs, depolarizing (300 ms) voltage steps from a holding potential of −80 from −40 to +50 mV in 10 mV steps (0.3 Hz) were applied. The pipette solution for the recording of I_Ca,L_ contained (in mM): 120 CsCl, 1 MgCl_2_, 5 Mg-ATP, 10 EGTA, and 5 Hepes (pH 7.4, CsOH). The extracellular solution was (in mM): 120 NaCl, 5 KCl, 3.6 CaCl_2_, 20 TEA-Cl, 1 MgCl, and 10 Hepes (pH 7.4, TEA-OH). Stimulation and inhibition of I_Ca,L_ are reported in terms of the percentage of the increase or decrease of I_Ca,L_ density, respectively. Only cells with a variation of I_Ca,L_ density of >5% from baseline were included into the statistics.

To record the time dependent, depolarization-activated outward K^+^ current (I_Ks_) the cells were perfused with an external solution containing the I_Kr_ blocker E4031 (1 µM), thereafter Isoproterenol (10 µM) was added and then Isoproterenol and the IKs specific blocker Chromanol (1 µM) were applied. I_Ks_ was elicited by a single 5 s lasting depolarizing voltage step to +50 mV from a holding potential of −40 mV [15]. The internal solution for the recording of I_K_ contained: 140 mM KCl, 4 mM MgATP, 5 mM EGTA, 1 mM MgCl_2_, and 10 mM HEPES (pH 7.4, KOH). The external solution contained: 140 mM *N*-methyl-Dglucamine, 5.4 mM KCl, 1 mM MgCl_2_, 0.1 mM CaCl_2_, 10 mM HEPES, 10 mM glucose (pH 7.2, HCl). Two mM 4-aminopyridine, 1 µM E 4031, 0.4 mM CdCl_2_, or 5 µM Nifedipine were added to block contaminating I_to_, I_Kr_, and I_Ca,L_, respectively. All recordings were performed at room temperature.

Recording of membrane potential was performed in the current clamp mode and with a pipette solution containing (in mM) 50 KCl, 80 K-Asparatate, 1 MgCl_2_, 3 MgATP, 10 EGTA, 10 HEPES, pH 7.4 (KOH) and an external solution containing 140 NaCl, 5.4 KCl, 1.8 CaCl_2_, 1 MgCl_2_, 10 HEPES, 10 glucose (pH 7.4, NaOH). The same solution was used to record Action Potential (AP), which were elicited by a 2.5 ms current injection pulse of 800–1000 pA though the patch pipette. APD at 90% of repolarization was analyzed with the cardiac action potential analysis module of LabChart. Data were acquired at a sampling rate of 10–20 kHz (voltage clamp) or 5 kHz (current clamp), filtered at 1 KHz, digitized with the Patchmaster software (HEKA, Germany) and analyzed offline using the Fitmaster (HEKA) or the LabChart software (AD Instruments, USA).

### Statistical analysis

Unless otherwise stated mean values with standard errors are shown. Statistical tests were performed using unpaired Student's t-Test for all data. A p-value of <0.05 was considered significant and is indicated by a * in the figures; ** corresponds to p<0.01, *** to p<0.001, ns to not significant.

## Results

### *Knock-out* of individual miR-1/133a clusters

**Figure 1:**
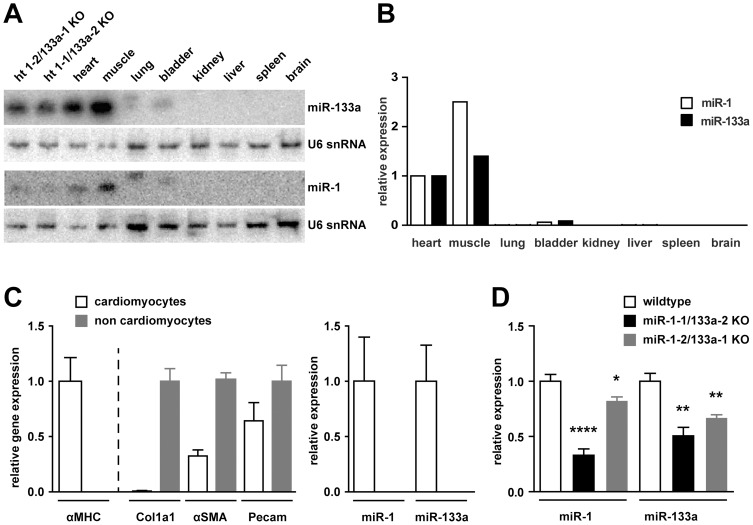
Expression of miR-1/133a in WT and mutant animals. Northern blot (A) as well as quantitative RT-PCR (B) detected the miRNAs miR-1 and miR-133a in total RNA isolated from heart (ht), skeletal muscle (m. tibialis anterior) and bladder. qRT-PCR analysis of isolated cardiomyocytes and non-cardiomyocytes (C). The identity of the fractions was confirmed by αMHC, collagen1a1, α-smooth muscle actin and Pecam/CD133 expression, respectively. Whereas the cardiomyocyte fraction contained some endothelial cells and possibly also smooth muscle cells, the non-cardiomyocyte fraction did not contain considerable amounts of cardiomyocytes. miRNA expression analysis demonstrates that expression of miR-1 and miR-133a is confined to cardiomyocytes (C). Deletion of single miR-1/133a clusters led to a reduced abundance of miR-1 or miR-133a in the heart detected by northern blot (A) or qRT-PCR (D).

Deletion of the miR-1/133a clusters from mouse chromosome 2 and 18 has been previously described [7]. Using northern blot analysis (Figure 1A) as well as qRT-PCR (Figure 1B) we demonstrate here tissue specific expression of miR-1 and miR-133a in adult heart, skeletal muscle and with reduced abundance in bladder. Isolation of cardiomyocytes and non-cardiomyocytes from cardiac tissue demonstrates that expression of both microRNAs is confined to cardiomyocytes (Figure 1C, n/group  = 4–6). Deletion of either miR-1/133a cluster resulted in significant reduction of miR-1 or miR-133a in the adult tissues. The qRT-PCR revealed that in adult heart tissue loss of the miR-1-1/133a-2 cluster leads to a significantly stronger reduction of miR-1 than deletion of the miR-1-2/133a-1 cluster (Figure 1D, n/group  = 4–6). The miR-1-2/133a-1 cluster is encoded in an intron of the protein coding gene Mib1. We found no difference in the expression of Mib1 between WT and miR-1-2/133a-1 *knock-out* animals (Figure S1, n/group  = 2–4), deletion of the miRNA cluster from the intron of the gene did not disturb the splicing or abundance of the Mib1 mRNA.

### Mice lacking a single miR-1/133a cluster develop normally and are vital

**Figure 2:**
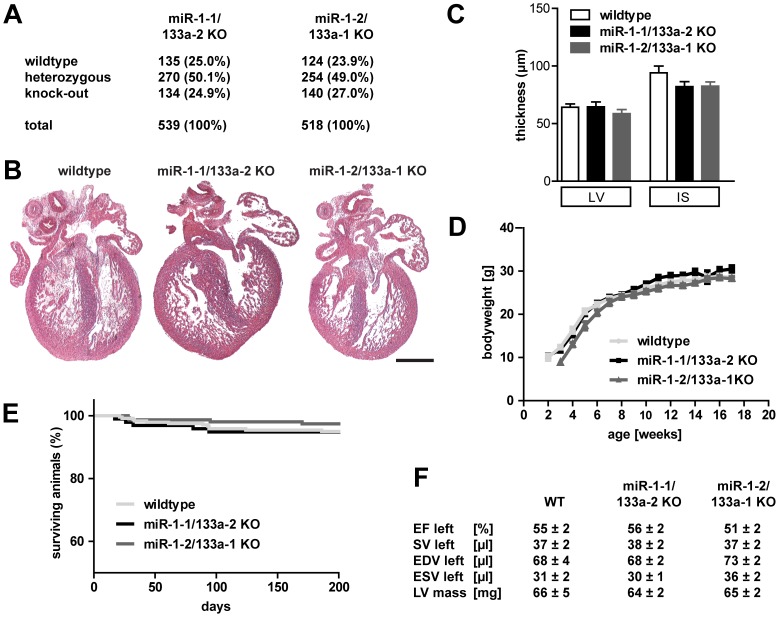
Loss of single cluster does not change critical heart function parameters. Deletion of a single miR-1/133a cluster did not impair embryonic survival, as indicated by the Mendelian distribution of genotypes after mating of heterozygous parents. The distribution was determined at the time of weaning (A).We did not observe morphological changes during heart development at E15.5, especially no ventricular septum defects are observed (B). At E15.5 the thickness of the left ventricular wall (LV) was not changed, similarly the thickness of the interventricular septum (IS) was not significantly reduced (C). Weight gain (D) and survival (E) of single cluster mutant animals was comparable to WT litter mate animals. Heart functional parameters were determined using MRI (F); ejection fraction (EF), stroke volume (SV), end-diastolic volume (EDV), end-systolic volume (ESV), left ventricular (LV) mass was analyzed and no significant differences in heart function were detected (F). The scale bar in B corresponds to 200 µm.

Breeding of the respective heterozygous animals in a 129/C57 mixed genetic background resulted in offspring with the expected Mendelian ratio, indicating that deletion of single miR-1/133a clusters did not cause embryonic lethality (Figure 2A). We analyzed the morphology of fetal hearts of single cluster *knock-out* animals at embryonic day 15.5 to screen for developmental defects (Figure 2B; n/group ≥10). We did not find changes in the histology of the heart at E15.5 and no changes in left ventricular wall or interventricular septum thickness were observed (Figure 2C, n/group ≥9). Loss of a single miR-1/133a cluster did not reduce the survival (n/group ≥97, log-rank test p>0.45) or the body weight development (n/group ≥6) of the respective *knock-out* animals compared to WT (Figure 2D, E). In addition, MRI analysis of heart structure and function of adult mice did not reveal obvious differences in any of the functional parameters (Figure 2F, n/group ≥7).

The miRNAs miR-1 and miR-133a are abundantly expressed in skeletal muscle. However, homozygous deletion of single miR-1/133a clusters yielded no striking changes in the morphology of skeletal muscle [7]. We did not observe alterations in fiber size distribution (Figure S2A; n/group ≥5) or changes in the number of centralized nuclei (Figure S2B). In both *knock-out* models we also did not observe differences in type 1 fiber content in the TA muscle (Figure S2C, D).

### Loss of single miR-1/133a clusters affects repolarization of the heart *in vivo*

**Figure 3:**
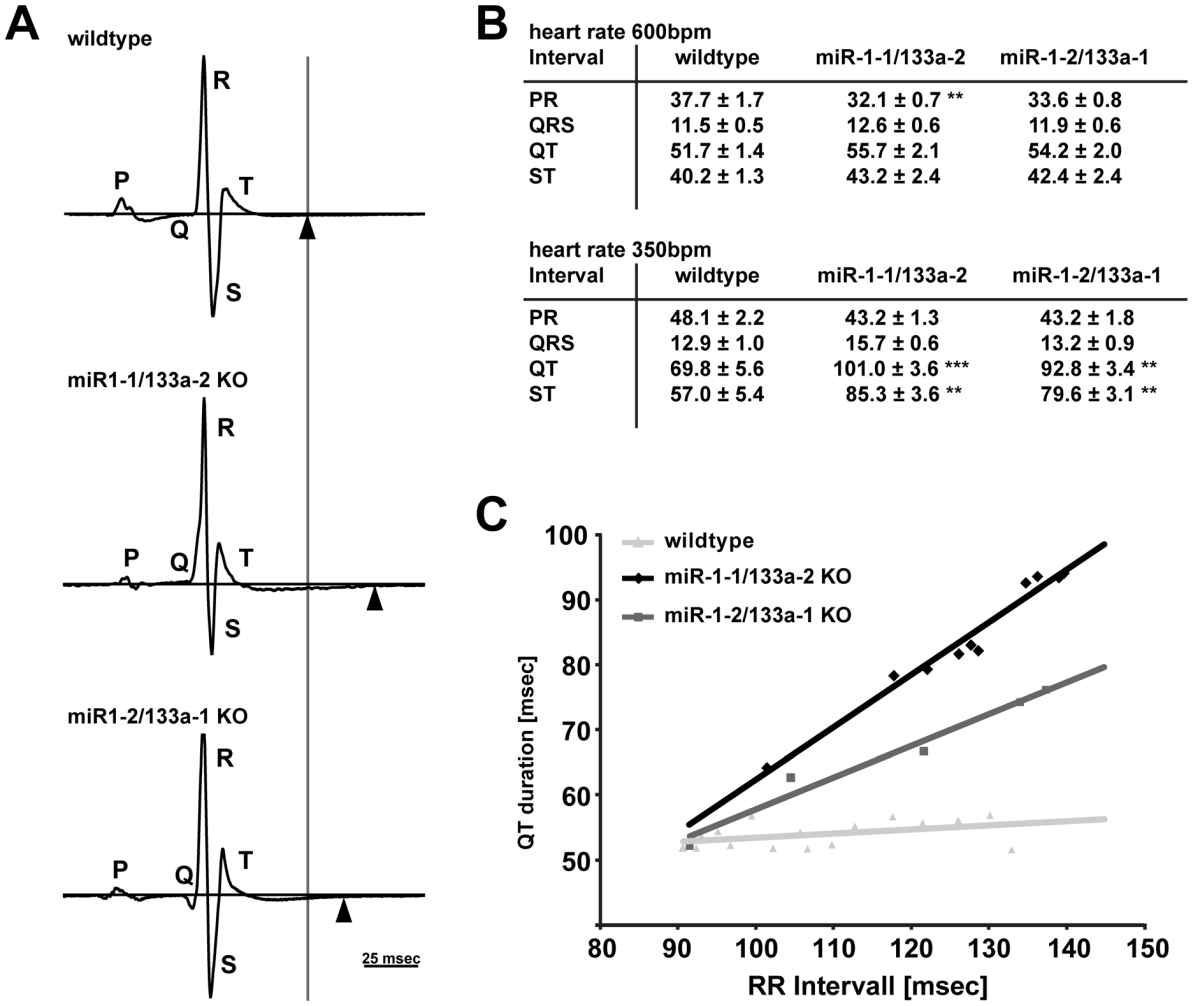
Loss of miR-1/133a impairs cardiac repolarization. Analysis of surface ECGs from immobilized animals revealed an increased QT duration (A). The increased QT duration was obvious especially at low heart rates (longer RR interval) induced by anesthesia with Isoflurane (B). We did not observe changes in PR or QRS interval length, nor arrhythmia or changes in the morphology of the ECG traces. The increased QT interval length is based on a longer ST duration. As shown in (C) the slope of a linear fit (ΔQT/ΔRR) is greater in miR-1/133a single cluster compared to the WT animals.

Electrocardiography was used to explore the electrophysiological consequences of loss of miR-1/133a clusters (Figure 3A). Many ECG parameters like PR- or QT-interval length strongly depend on the heart rate, thus in humans heart rate corrected parameters like the QTc are used. The algorithms used for calculation of human ECG parameters might not be appropriate for the mouse model [16], therefore ECGs were acquired at different heart rates and were analyzed dependent on heart rate or the RR interval length, respectively. ECGs were acquired using a custom made amplifier attached to a device to immobilize animals for the recording time that also allows anesthesia to manipulate the heart rate. Analysis of ECGs of our respective single miR-1/133a cluster KO mice revealed no change in the morphology of the R wave in any of our models (Figure 3A). Also we did not observe changes in QRS complex length at different heart rates indicating that conduction and depolarization of the heart is apparently not changed (Figure 3B). No arrhythmias were detected in the ECGs of our *knock-out* models. However, in both KO models we consistently detected a prolonged QT interval compared to control mice of the same age and this prolongation was found to be more pronounced at lower heart rates (Figure 3B), as also indicated by statistical analysis of ΔQT/ΔRR (Figure 3C) for the *knock-out* mice (ΔQT/ΔRR in WT: 0.27±0.08, in miR-1-1/133a-2: 0.63±0.04 p<0.01, in miR-1-2/133a-1: 0.57±0.06 p<0.01; n = 18/10/14). Prolonged QT durations (93.0±5.0 ms at heart rate 350 bpm, ΔQT/ΔRR: 0.45±0.09 p<0.05 compared to WT) were also observed in miR-1-2/133a-1^del^ mutant mice after deletion of the neomycin resistance cassette (n = 11), indicating that the increased QT duration always depends on the loss of miR-1/133a, but not on potential disturbance of neighboring genes. Of note the extension of the QT interval was always based on a longer ST duration with unchanged QRS duration.

In addition to ECG recordings with immobilized mice we also performed telemetric recording of ECG using implanted transmitters. Heart rates were not significantly different between WT and mutant animals with higher mean heart rates at night than at day. We took advantage of the variation of the heart rate from 400 bpm up to 650 bpm during circadian rhythm to acquire the QT interval length at different heart rates in non-sedated mice. Also in this freely moving mice we observed a significantly longer QT interval at lower heart rates in the miR-1/133a mutant mice compared to WT mice (ΔQT/ΔRR; n/group 2–5, WT: 0.27±0.03, miR-1-1/133a-2: 0.57±0.02, p<0.01, miR-1-2/133a-1: 0.89±0.11, p<0.001). ECG data obtained by telemetric recording were screened for Torsades de pointes that potentially might occur due to prolonged QT duration. No such events were detected in more than 30 h of ECG recordings obtained from 5 different mutant mice. Although we never observed death of animals during telemetric recording of ECG, the unchanged survival of mutant *knock-out* animals supported the view that fatal Torsades de pointes arrhythmia did not occur in miR-1/133a KO mice.

### Molecular consequences of loss of single miR-1/133a clusters

**Figure 4:**
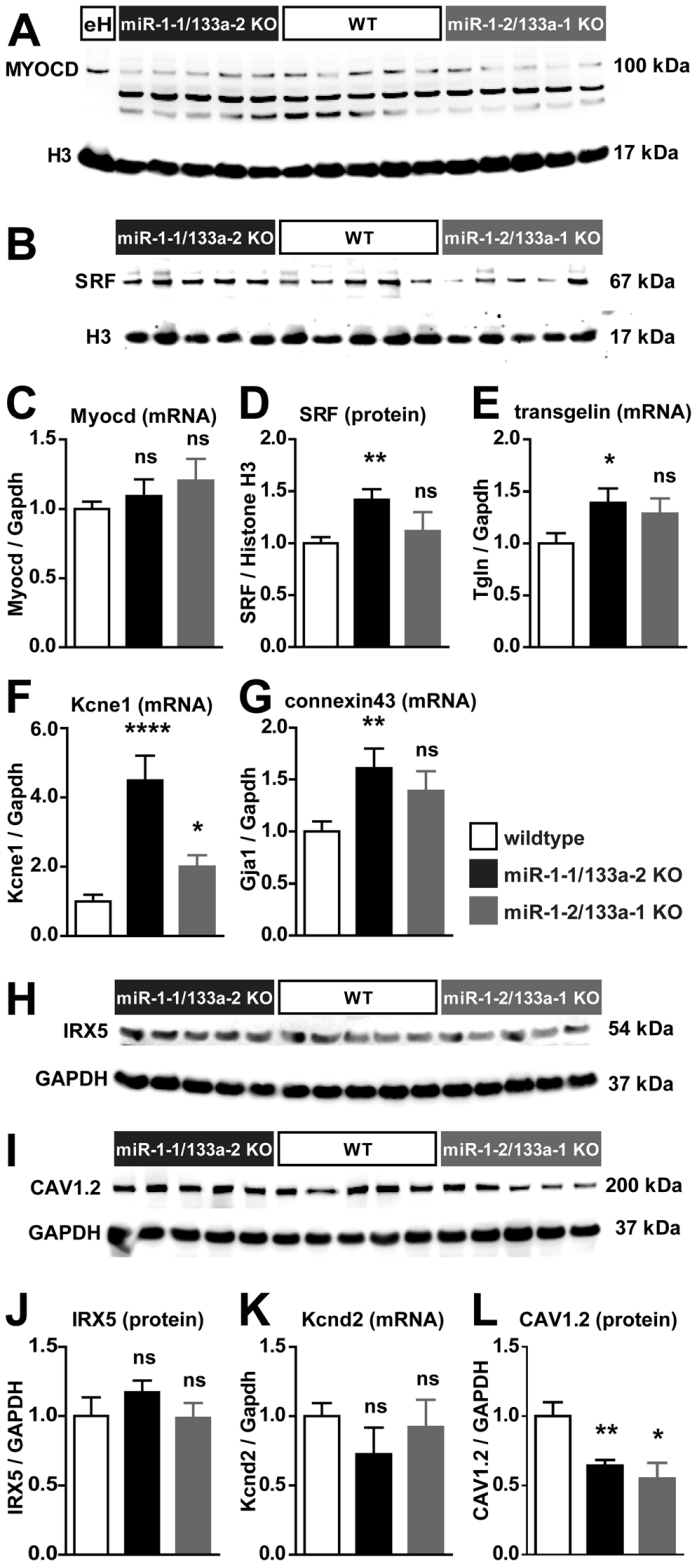
Molecular changes induced by reduced miR-1/133a abundance. mRNA as well as protein expression of several previously described direct miR-1 or miR-133a target molecules was analyzed in heart tissue of WT and respective single miR-1/133a cluster knock-out animals to validate the regulation of these targets in our in vivo models. In the heart of single cluster knock-out animals the miR-1 target myocardin was not upregulated at protein level (A) or mRNA level (C). This applied to the embryonic heart (eH) specific isoform of myocardin as well as to the other isoforms exclusively detected in the adult heart. In contrast the miR-133a target SRF is significantly upregulated in the heart of miR-1-1/133a-2 knock-out mice (B, D). In line with up-regulation of SRF, we also confirmed the Affymetrix-analysis based finding that smooth muscle marker genes were upregulated in the adult heart of miR-1-1/133-2 mice (E). We detected significant upregulation of the miR-1 target Kcne1 in both single cluster knock-out (F). The miR-1 target connexin43 was significantly upregulated in only miR-1-1/133a-2 mutant hearts, corresponding to the more severe loss of miR-1 in this model (G). We did not find upregulation of the previously described miR-1 target IRX5 (H, J). qRT-PCR analysis confirmed the unbiased transcriptome analysis based finding that the proposed Irx5 transcriptional target Kncd2 is not significantly regulated in mutant hearts (K). We observed a downregulation of the previously described miR-1 target gene CAV1.2 (I, L).

To get insights into the molecular mechanisms leading to the phenotypic changes observed after loss of single miR-1/133a clusters we analyzed transcriptional changes in WT vs. the respective miR-1/133a *knock-out* hearts. Microarray analysis was performed using RNA isolated from whole hearts excluding atria of 7 WT, 4 miR-1-1/133a-2 and 5 miR-1-2/133a-1 KO animals. We only found few transcriptional changes in molecules previously described to be direct targets of miR-1 or miR-133a regulation (Table 1). In addition to unbiased transcriptome analysis we extended our analysis of mRNA expression using quantitative RT-PCR (Figure 4C, E–G, K) with higher number of independent replicates or performed western blot analysis of protein expression when applicable (Figure 4A, B, H, I, n/group  = 5–9). Interestingly, we observed activation of a smooth muscle gene program in the miR-1/133a *knock-out* mice (Acta2, calponin, transgelin, Myl9, Myh11; table 1). In E10.5 embryonic hearts deletion of both miR-1/133a clusters resulted in upregulation of myocardin at the RNA, as well as at the protein level [7]. However, analysis of myocardin protein expression in the adult heart in contrast to protein extracts from embryonic hearts revealed signals of different molecular weight, which may be corresponding to splice variants of myocardin. None of the detected signals indicated increased protein abundance in adult heart (Figure 4A). In addition, in adult hearts upregulation of myocardin at the transcript level also was not evident (Figure 4C). At the protein level we observed upregulation of the miR-133a target gene SRF in the miR-1-1/133a-2 mutant mice, but not in the miR-1-2/133a-1 mutant mice (Figure 4B, D). This upregulation of SRF correlates well with the observed changes in smooth muscle genes (Table1, Figure 4E).

**Table 1:** Transcriptome analysis after deletion of single miR-1/133a clusters.

Fold change miR-1-1/133a-2 vs. WT	Fold change miR-1-2/133a-1 vs. WT	p-value miR-1-1/133a-2 vs. WT	p-value miR-1-2/133a-1 vs. WT	Gene symbol	Gene title	comment
2,15	2,35	0,027	0,025	Myh7	myosin heavy chain β	
2,10	1,68	0,015	0,003	Acta2	smooth muscle alpha 2 actin	sm
1,84	1,13	0,005	0,466	Myl9	myosin regulatory light chain 9	sm
1,67	0,88	0,023	0,409	Cnn1	calponin 1	sm
1,64	0,91	0,003	0,570	Tagln	transgelin	sm
1,59	1,41	0,027	0,057	Kcne1	K+ v.-gated channel, Isk-rel. subfamily, member 1	t
1,56	1,06	0,010	0,770	Myh11	smooth muscle myosin heavy chain 11	sm
1,31	1,28	0,211	0,065	Irx5	Iroquois related homeobox 5	t
1,31	1,14	0,038	0,127	Myocd	myocardin	t
1,27	1,21	0,167	0,169	Gja1	gap junction protein, α 1	t
1,05	0,98	0,659	0,886	Srf	serum response factor	t
1,04	1,23	0,656	0,042	Cacna1c	L-type calcium channel, α 1C subunit	t
0,90	0,92	0,473	0,498	Kcnd2	K+ v.-gated channel, Shal-rel. family, member 2	

Affymetrix GeneChip transcriptome analysis after loss of single miR-1/133a clusters revealed only limited regulation of miR-1/133a target molecules. Of note Kcne1 was upregulated in both *knock-out* models, however many other previously described miR-1 or miR-133a targets were not regulated after loss of roughly 50% miR-1 or miR-133a. Consistent with upregulation of SRF in miR-1-1/133a-2 mutant animals we observe upregulation of smooth muscle/fetal gene program markers in these mutants. Kcnd2 has been described to be a direct transcriptional target of Irx5. The comment indicates that the gene belongs to the smooth muscle gene program (sm) or is a predicted target of miR-1/133a regulation (t).

Clearly, we observed upregulation of the miR-1 target gene Kcne1 in the miR-1/133a *knock-out* mice and confirmed that by qRT-PCR (Figure 4F). Microarray analysis detected no significant upregulation of the previously described miR-1/133a targets connexin43/Gja1, Irx5 or Cav1.2, but qRT-PCR analysis with higher n-number revealed increased abundance of connexin43 by qRT-PCR in miR-1-1/133a-2 mutant mice (Figure 4G). In contrast to previous observations we did not observe an increase of IRX5 protein abundance in either of the mouse models (Figure 4H, J). Irx5 has been suggested to be a direct transcriptional regulator of Kcnd2 and thus causal for the arrhythmias and QRS-complex malformations in the miR-1-2 *knock-out* mice [2], therefore we also analyzed the expression of Kcnd2 transcripts in our mouse models. We did not detect regulation of Kcnd2 in the microarray experiments nor by quantitative RT-PCR (Figure 4K). Cav1.2 has been described to be a miR-1 target molecule in rat and human [17]. However, the described miR-1 target sites are not conserved in the 3′UTR of mouse Cacna1c. In addition we did not detect upregulation of Cav1.2 at the protein level (Figure 4I, L) as one would predict for a miRNA target molecule after deletion of the miRNA. We detected downregulation of the CAV1.2 calcium channel that is essential for depolarization of the cardiomyocyte membrane potential during excitation, indicating secondary regulatory events.

### Changes in electrophysiological properties of cardiomyocytes

**Figure 5:**
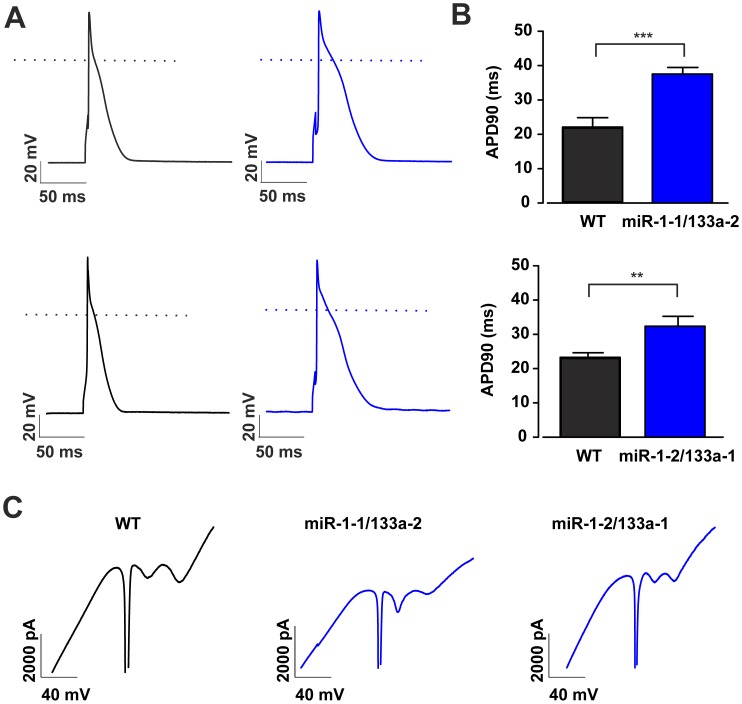
Increased Action Potential Duration at 90% of repolarization (APD90) in miR-1/133a knock-out ventricular cardiomyocytes. Action potential (AP) traces in isolated adult ventricular cardiomyocytes from the respective miR-1/133a cluster knock-out animals (blue) and the corresponding controls (black). APs were elicited by 2.5 ms lasting current injections of 800–1000 pA through the patch pipette (A). Statistical analysis of APD90 in miRNA control (WT) and respective miR-1/133a knock-out cells (B). Representative ramp depolarizations (−150 mV to +60 mV, 250 ms; holding potential −80 mV) recorded from WT and miR-1/133a KO ventricular cardiomyocytes (C) prove the functional expression of the most important ion currents.

To understand the cellular basis for the observed ECG changes and to identify the underlying mechanisms we performed single cell patch clamp analysis on adult isolated ventricular cardiomyocytes from both miR-1/133a cluster *knock-out* animals and respective littermate controls. First, we determined action potential duration at 90% of repolarization (APD_90_); APs were evoked in the current clamp mode by injecting small depolarizing currents (Figure 5A). The cardiomyocytes displayed very similar resting membrane potentials (for miR-1-1/133a-2 control cells −82.2±0.8 mV, n = 33, miR-1-1/133a-2 mutant cells −82.3±0.8 mV, n = 39; for miR-1-2/133a-1 control cells −81.6±1.3 mV, n = 31, miR-1-2/133a-1 KO cells −80.9±0.9 mV, n = 29). However, we found in both miR-1/133a mutants a significantly prolonged APD_90_, as would be expected from the ECG data (for miR-1-1/133a-2 control cells 21.9±2.9 ms, n = 33, for miR-1-1/133a-2 KO cells 37.6±1.8 ms, n = 39, p<0.001; for miR-1-2/133a-1 control cells 23.2±1.4 ms, n = 31, for miR-1-2/133a-1 KO cells 32.4±2.8 ms, n = 29, p<0.01; Figure 5B). The prolongation of the APD_90_ was more pronounced in the miR-1-1/133a-2 KO cells, which was fully in agreement with the observed changes in the ECG in the respective mice. We next explored, whether prominent differences in voltage dependent ion currents could be detected by applying ramp depolarizations (from −150 to +60 mV, 250 ms). These experiments yielded similar inward and outward current components for both miR-1/133a *knock-out* models and the respective control groups (Figure 5C), proving the functional expression of the most important ion currents.

One of the potential targets of the two miRNAs of interest was found to be KCNE1 [18] that together with KCNQ1 constitutes the I_Ks_ repolarizing current. We found Kcne1 upregulation in microarray experiments and qRT-PCR, however we did not detect KCNE1 protein expression in adult wildtype or miR-1/133a *knock-out* mouse cardiomyocytes. This is in accordance with previous reports showing that I_Ks_ and I_Kr_ are diminished in adult mouse cardiomyocytes and apparently do not play an important role for the repolarization of the cardiac AP [19], although interestingly loss of function of Kcne1 results in modulation of QT adaptation at heart rate variations in mice [16]. Moreover re-expression of a functional I_Ks_ in adult mouse cardiomyocytes would apparently favor repolarization and therefore rather cause shortening of the APD. Nevertheless, we measured the repolarizing I_Ks_ in single cardiomyocytes using well established protocols [15]. As a positive control I_Ks_ was measured in embryonic WT cardiomyocytes (E14.5–E16.5), in which I_Ks_ is known to be functionally expressed (Figure S3A–B). Then, we determined whether I_Ks_ was increased in miR-1-2/133a-1 mutant cells. Despite the observed transcriptional Kcne1 upregulation, we could not detect activation of macroscopic I_Ks_ neither in miR-1-2/133a-1 control (n = 3) nor KO cardiomyocytes (n = 13; Figure S3C, D), fully in line with the proposed lack of function of this K_V_ component in the adult mouse heart. Thus, our data demonstrate that differences in the functional expression of I_Ks_ do not underlie the observed prolongation of the APD_90_.

Besides I_Ks_, also other outwardly rectifying I_K_ such as I_to_ and I_Kr_ could be responsible for the AP prolongation. Indeed the molecular basis of I_to_ is Kcnd2/Kv4.2 that has previously been claimed to be transcriptionally regulated by the miR-1 target Irx5 and to be at least in part responsible for cardiac conduction defects observed in miR-1-2 mutant mice [2]. That mechanism has not been confirmed in miR-1-1 mutant mice [5] and we did not see regulation of Kcnd2 transcripts in our models (Figure 4K). Nevertheless we investigated these I_K_ components in adult cardiomyocytes in the current clamp mode without and in presence of the K^+^-channel blocker 4-AP (2 mM; Figure S4A–B) [19]–[21]. We reasoned that strong differences in the expression of these outward rectifying I_K_ should yield drastic differences in the 4-AP effect on APD_90_. This was not the case (Figure S4C), as the 4-AP-induced prolongation of the APD_90_ was relatively similar in miR-1/133a KO cardiomyocytes compared to the respective control cardiomyocytes, indicating that the observed ECG changes are unlikely due to differences in these I_K_ components.

**Figure 6:**
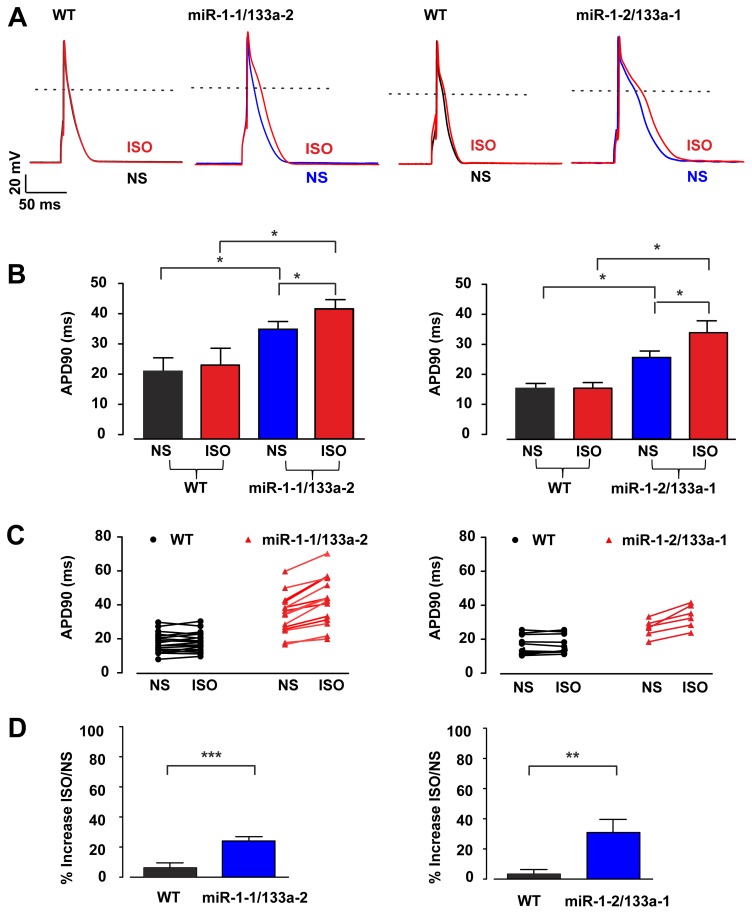
ß-adrenergic signaling prolongs APD90 in miR KO ventricular cardiomyocytes. Representative APs of control (black traces) and KO cells without (blue traces) and in presence of Isoproterenol (ISO; 1 µM, red traces) (A). Statistics of APD90 in miR WT and KO cells without and with ISO (B). Point graphs of APD90 of individual cells prior and after Isoproterenol application underscores the different response of control and miRNA KO cells (C). Percentage of increase of APD90 in miR control and KO cells (D).

**Figure 7:**
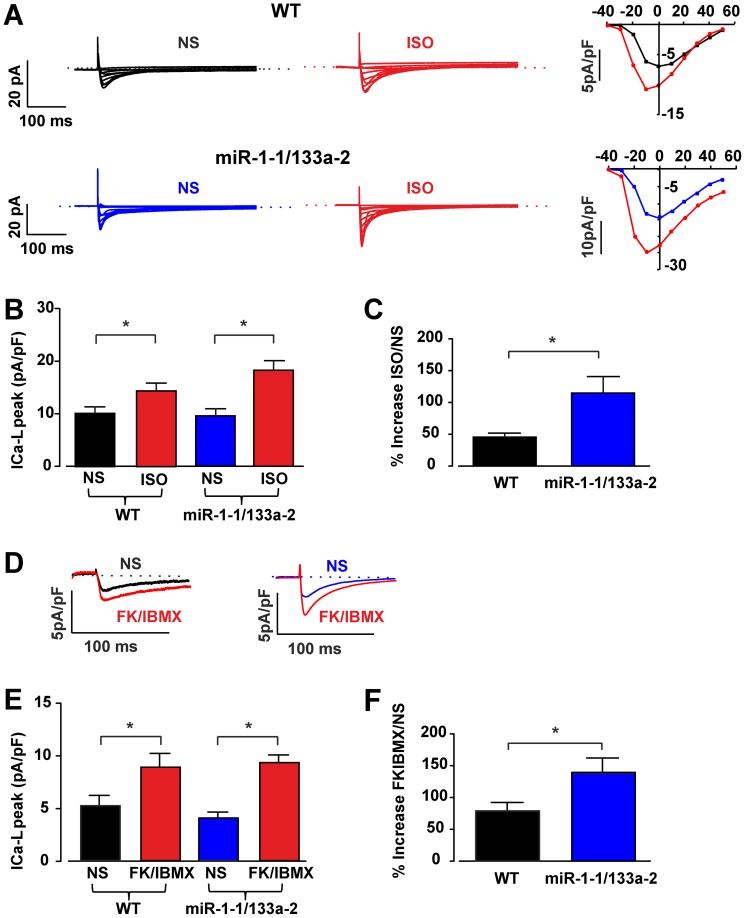
ß-adrenergic stimulation leads to a stronger stimulation/recruitment of ICa,L in miR-1-1/133a-2 KO ventricular cardiomyocytes. Representative IV-curves of ICa,L in miR-1-1/133a-2 control (upper traces) and KO cells (lower traces); IV curves of ICa,L were elicited by applying 300 ms long depolarizing voltage steps from a holding potential of −80 mV to voltages ranging from −40 mV to +50 mV in 10 mV steps in normal solution (left traces) and upon application of Isoproterenol (ISO; 1 µM, red traces) (A). Bar graph of the % of ISO-induced stimulation of ICa,L density in miR-1-1/133a-2 control and KO cells (B, C). Representative traces of miR-1-1/133a-2 control (left) and KO (right) ventricular cardiomyocytes recorded in normal solution (black or blue, respectively) and upon maximal stimulation by the combined application with Forskolin (FK, 10 µM) and IBMX (100 µM) (red) (D). Percentage of stimulation of ICa,L by FK and IBMX (E, F).

Besides the expression of ion channels also changes in their modulation, in particular by hormones of the autonomous nervous system, could underlie the observed changes in the electrical features and APD prolongation. We therefore explored the effect of the adrenergic agonist Isoproterenol (1 µM) on the APs. We found that Isoproterenol application led to a clear and significant prolongation of the APD_90_ in all miR-1-2/133a-1 and miR-1-1/133a-2 KO cardiomyocytes (Figure 6A–C). APD_90_ in the miR-1-1/133a-2 control cardiomyocytes was 22.2±1.5 ms in normal solution and 24.1±5.6 ms (n = 18) upon Isoproterenol application, whereas in miR-1-1/133a-2 KO cardiomyocytes it increased from 35.8±2.6 ms to 42.6±3.1 ms in the presence of Isoproterenol (n = 19). Similarly, in miR-1-2/133a-1 control cells the APD_90_ was 16.6±1.7 ms in normal solution and 16.9±1.6 ms (n = 10), in presence of Isoproterenol, whereas in miR-1-2/133a-1 KO cells it was 26.8±2.3 ms and 35.2±4.2 ms (n = 6) in normal solution and Isoproterenol, respectively. The percentage of Isoproterenol stimulation in respect to the normal solution was for miR-1-2/133a-1 3.5±2.9% in control cells and 30.9±8.7% in KO cells (p = 0.003) and for miR-1-1/133a-2 control cells 6.5±3.1% (n = 18) and KO cells 24.2±2.5% (n = 15; p = 0.0002), respectively (Figure 6D). Since the β-adrenergic agonist Isoproterenol is involved in the modulation of the L-type calcium current (I_Ca,L_) and this channel is also implicated in some LQT related mutation [22], [23] we next investigated the expression of this current in miR-1-1/133a-2 control and KO animals using voltage clamp protocols. Due to of the known modulation of I_Ca,L_ by adrenergic signaling and potential differences in the phosphorylation status at rest, we analyzed I_Ca,L_ in IV-curves without and in presence of Isoproterenol (1 µM) (Figure 7A). The IV curves revealed a similar voltage dependence for peak I_Ca,L,_ which was close to 0 mV (−3.0±1.5 mV and −2.9±1.8 mV, for both control and miR KO cardiomyocytes, respectively, n = 7) and shifted to a more negative potential close to −10 mV (−9.0±1.2 and −8.6±1.4 mV in control and KO cardiomyocytes, respectively, n = 7) upon Isoproterenol application. In addition, we also performed experiments, where I_Ca,L_ density was measured at basal conditions and upon maximal stimulation by combined application of the direct adenylate cyclase-activator Forskolin (FK, 10 µM) and the phosphodiesterase-inhibitor IBMX (100 µM) (Figure 7D). These experiments showed that I_Ca,L_ could be stimulated either by Isoproterenol (miR-1-1/133a-2 control cells 45.4±4.2%, n = 14, miR-1-1/133a-2 KO cells 114.9±22.5%, n = 11, p = 0.012) or FK/IBMX (miR-1-1/133a-2 control cells 79.7±12.6%, n = 10, miR-1-1/133a-2 KO cells 140.2±22.1%, n = 13, p = 0.028) significantly stronger in the miR-1-1/133a-2 KO cardiomyocytes, implying differences in the phosphorylation levels (Figure 7B–C, E–F). Importantly, we did not observe significant differences in inactivation kinetics of I_Ca,L_ between WT and KO cardiomyocytes (data not shown). Similar to the current density analysis, the analysis of the total I_Ca,L_ charge influx showed no differences under basal conditions but upon stimulation with Isoproterenol we observed significant larger charge influx in KO cardiomyocytes compared to WT cardiomyocytes (p = 0.02, data not shown).

As reported above in the ECG evaluation, the LQT-phenotype became particularly obvious at low frequencies of the heart, which is compatible with LQT-syndromes due to alterations of voltage dependent-Na^+^ (LQT 3) [24] or Ca^2+^ (LQT 8) channels [22], [23], whereas LQT-syndromes related to K^+^-channels (LQT1 and LQT2) become rather symptomatic at high heart rates [25], [26]. Besides from I_Ca,L_ we therefore analyzed also key electrophysiological parameters of I_Na_, which are known to be altered in LQT3 with a prominent prolongation of APD and life threatening arrhythmias preferentially at night albeit at low heart rates. We focused on I_Na_ in the miR-1-2/133a-1 KO line (Figure S5A–D). The peak density of I_Na_ in cardiomyocytes from miR-1-2/133a-1 control and KO mice was very similar (for control cells 19.4±2.6 pA/pF, n = 15, for KO cells 17.2±2.2 pA/pF, n = 23) (Figure S5A, B). Also the recovery from inactivation of I_Na_ was found to be in a similar range (time constant τ in control cells 6.8±1.5 ms, n = 14, in KO cells 6.7±0.7 ms, n = 22) in control and KO cardiomyocytes (Figure S5C, D), indicating that typical changes of I_Na_ as found in LQTS 3, namely reduced density of I_Na_ and faster recovery from inactivation [24], are not responsible for the observed phenotype.

Thus, our electrophysiological evidence implies differences in the activity levels of I_Ca,L_, but not I_Na_. Defective regulation of voltage dependent Ca^2+^ channels would be consistent with the observed prolongation of the APD_90_ as well as with the increased QT-duration at low heart rates. Indeed, increased I_Ca_ also led to an increased APD_90_ in an *in silico* model simulating ion currents and Ca2+ fluxes of mouse ventricular cardiomyocytes (+ 50% I_Ca,L_  =  +41% APD_90_; +100% I_Ca,L_  = +108% APD_90_) [27].

### Molecular analysis of adrenergic signaling

**Figure 8:**
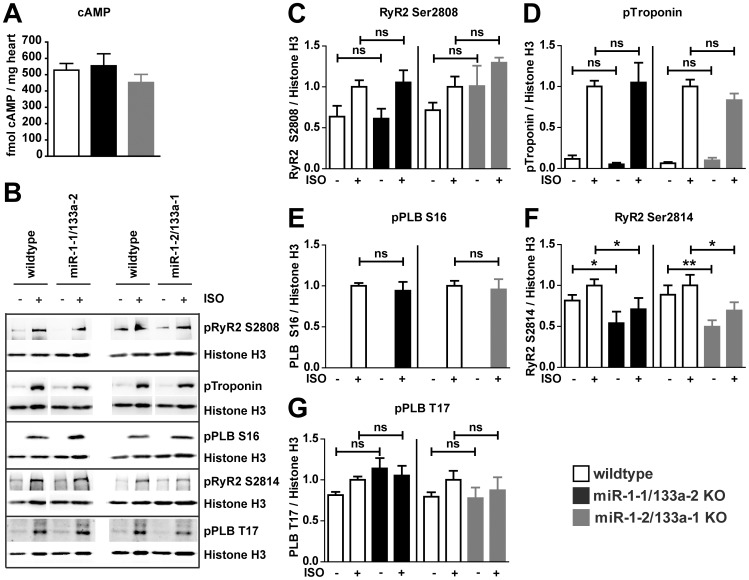
β-adrenergic signaling is intact in after loss of miR-1/133a. The concentration of cAMP was not changed in the adult heart of miR-1/133a single cluster mutant mice (A). Adrenergic signaling of cardiomyocytes isolated form adult hearts of WT and single miR-1/133a cluster mutant mice was investigated by stimulation with 1 µM Isoproterenol (ISO; B–G). Adrenergic signaling affects multiple components involved in cardiomyocyte calcium handling and contraction. Phosphorylation of several targets of the adrenergic signaling cascade was analyzed to detect modulation of the adrenergic signaling in cardiomyocytes isolated from mutant and WT animals. Proteins of cardiomyocytes isolated from of ≥5 animals was used for statistical evaluation (C–G), representative blots are shown (B).

Analysis of the electrophysiological properties of cardiomyocytes isolated from mutant mice indicated modulation in the adrenergic control of calcium channel activity upon reduced miR-1/133a expression in the single cluster mutant mice. Unbiased transcriptome analysis did not reveal prominent changes in the expression of components of the adrenergic signaling cascade that may be target of miR-1 or miR-133a regulation (Table 2). Stimulation of β-adrenergic receptors results in G-protein mediated activation of adenylatecyclase, subsequently cAMP formation and activation of PKA or of the calcium/calmodulin-dependent protein kinase II (CaMKII) that participates to mediate effects of β-adrenergic signaling on calcium handling [28]. To get insights into the activity of these cascades we first analyzed cAMP concentrations in mutant vs. WT hearts. No changes in the cAMP concentration could be detected (Figure 8A, n/group  = 4–5). To reveal changes in adrenergic signaling that could potentially influence plasma-membrane calcium channel activity and hence QT duration, we isolated cardiomyocytes from WT and the respective *knock-out* mice and analyzed known phosphorylation target sites of the β-adrenergic signaling. Unfortunately, the phosphorylation the Cav1.2 calcium channel cannot reliably be accessed due to lack of appropriate antibodies. Therefore we analyzed the activation of other cellular targets of the adrenergic signaling by comparison of Isoproterenol-stimulated vs. unstimulated cardiomyocytes isolated from WT or respective mutant mice. Isoproterenol treatment was effective to stimulate phosphorylation of known elements of the β-adrenergic signaling cascade (Figure 8B–G, n/group  = 6–7). However, none of the PKA dependent phosphorylation sites analyzed (RyR S2808, Troponin S23/24, PLN S16, Figure 8C–E) occurred to be changed in its responsiveness to Isoproterenol stimulation when we compared the mutant cardiomyocytes to the respective WT controls. Interestingly the CaMKII-dependent [28] phosphorylation of RyR2 S2814 (Figure 8F) is consistently decreased in miR-1-1/133a-2 and miR-1-2/133a-1 *knock-out* cardiomyocytes compared to cardiomyocytes isolated from WT mice, however the CaMKII dependent Phospholamban (PLB) T17 phosphorylation site was not differently regulated (Figure 8G). To supplement the unbiased transcriptome analysis, we analyzed protein expression of molecules potentially modifying L-type calcium channel activity (Figure S6). The analysis of protein expression confirmed our transcriptome analysis. The previously identified miR-1 target B56α was not upregulated at transcript-level nor on protein level and the miR-1 target Sorcin (SRI) was significantly more abundant only in miR-1-1/133a-2 mutant hearts. In addtion the CAVB2 subunit of the L-type calcium channel was not differentially regulated in WT and mutant hearts. Taken together these data indicate a regulation of particular aspects of β-adrenergic signaling in miR-1/133a *knock-out* cardiomyocytes, may be by modulatory factors acting downstream in the adrenergic signaling cascade.

**Table 2:** Potential miR-1/133a targets involved in modulation of adrenergic signaling.

Fold change miR-1-1/133a-2 vs. WT	Fold change miR-1-2/133a-1 vs. WT	p-value miR-1-1/133a-2 vs. WT	p-value miR-1-2/133a-1 vs. WT	Gene symbol	Gene title	predicted target of miRNA
1,07	1,03	0,460	0,411	Adcy1	adenylate cyclase 1	miR-133a (ts)
1,12	0,98	0,006	0,701	Adcy5	adenylate cyclase 5	miR-133a (ts)
1,07	1,03	0,605	0,683	Adcy6	adenylate cyclase 6	miR-133a (ts)
1,14	0,94	0,167	0,457	Adcy7	adenylate cyclase 7	miR-133a (ts)
1,17	0,99	0,143	0,945	Adora1	adenosine A1 receptor	miR-133a (ts)
0,99	0,96	0,919	0,620	Adra1a	adrenergic receptor, α 1a	miR-133a (ts)
0,98	1,11	0,846	0,254	Adra1b	adrenergic receptor, α 1b	miR-1 (ts)
1,03	0,96	0,479	0,432	Adra2a	adrenergic receptor, α 2a	miR-133a (ts)
0,93	0,89	0,610	0,177	Adrbk2	adrenergic receptor kinase, beta 2	miR-1 (ts)
1,11	1,14	0,244	0,118	Akap12	A kinase (PRKA) anchor protein (gravin) 12	miR-133a (ts, m)
1,11	1,08	0,142	0,249	Akap13	A kinase (PRKA) anchor protein 13	miR-1 (ts)
1,17	1,05	0,096	0,459	Akap2	A kinase (PRKA) anchor protein 2	miR-1 (ts)
1,12	1,01	0,095	0,876	Akap5	A kinase (PRKA) anchor protein 5	miR-133a (ts)
1,03	1,04	0,698	0,506	Akap8	A kinase (PRKA) anchor protein 8	miR-133a (ts)
1,05	1,04	0,203	0,463	Akap9	A kinase (PRKA) anchor protein (yotiao) 9	miR-133a (ts)
1,01	1,05	0,925	0,493	Atp2a2	Ca-ATPase, cardiac muscle, slow twitch 2	miR-133a (ts)
0,97	1,07	0,684	0,452	Calm2	calmodulin 2	miR-1 (ts)
1,09	1,03	0,415	0,566	Camk2a	Ca/calmodulin-dependent protein kinase II α	miR-1 (m)
0,72	0,77	0,029	0,162	Camk2d	Ca/calmodulin-dependent protein kinase II, delta	miR-133a (ts)
1,02	1,12	0,812	0,056	Gnai1	guanine nucleotide binding protein, α inhibiting 1	miR-133a (ts)
1,02	1,02	0,705	0,470	Gnai3	guanine nucleotide binding protein, α inhibiting 3	miR-133a (ts)
1,23	1,16	0,071	0,064	Gnb1	guanine nucleotide binding protein, beta 1	miR-1 (ts)
1,05	1,02	0,339	0,774	Gng12	guanine nucleotide binding protein, γ 12	miR-1 (ts)
1,02	1,00	0,833	0,988	Gng4	guanine nucleotide binding protein, γ 4	miR-1/133a (m)
1,08	1,12	0,092	0,062	Gng7	guanine nucleotide binding protein, γ 7	miR-1 (ts)
1,04	1,04	0,379	0,375	Pde4b	phosphodiesterase 4B, cAMP specific	miR-1 (ts, m)
0,98	0,99	0,659	0,687	Pde6a	phosphodiesterase 6A, cGMP-specific, rod, α	miR-133a (ts)
0,77	0,75	0,045	0,017	Pde7a	phosphodiesterase 7A	miR-1 (ts, m)
1,02	1,05	0,797	0,224	Pde8b	phosphodiesterase 8B	miR-1 (ts, m)
1,06	0,89	0,559	0,091	Pla2g4a	phospholipase A2, group IVA	miR-1 (ts, m)
1,12	1,02	0,190	0,751	Plcb2	phospholipase C, β 2	miR-133a (ts)
1,08	1,08	0,318	0,374	Ppp2ca	protein phosphatase 2, catalytic subunit, α isoform	miR-133a (ts)
1,14	1,05	0,272	0,367	Ppp2r5a	protein phosphatase 2, regulatory subunit B (B56), α	miR-1 (ts)
0,94	1,01	0,603	0,854	Prkaa2	protein kinase, AMP-activated, α2 catalytic subunit	miR-1 (ts, m)
1,09	1,17	0,178	0,006	Prkacb	protein kinase, cAMP dependent, catalytic, β	miR-1 (ts)
1,21	1,03	0,183	0,792	Slc8a1	solute carrier family 8, member 1	miR-1 (ts)
1,22	1,00	0,031	0,988	Sri	sorcin	miR-1 (ts, m)

Molecules known to be involved in adrenergic signaling as well as functionally related molecules were analyzed for potential targeting by mmu-miR-1 or mmu-miR-133a using Targetscan (ts) and mirBase.org (m) and transcriptional regulation of these molecules was analyzed. Of the 177 molecules analyzed to be related to adrenergic signaling 39 were predicted potential targets of miR-1 or miR-133a.

### miR-1/133a regulates the impact of adrenergic signaling on L-type calcium channel activity *in vivo*

**Figure 9:**
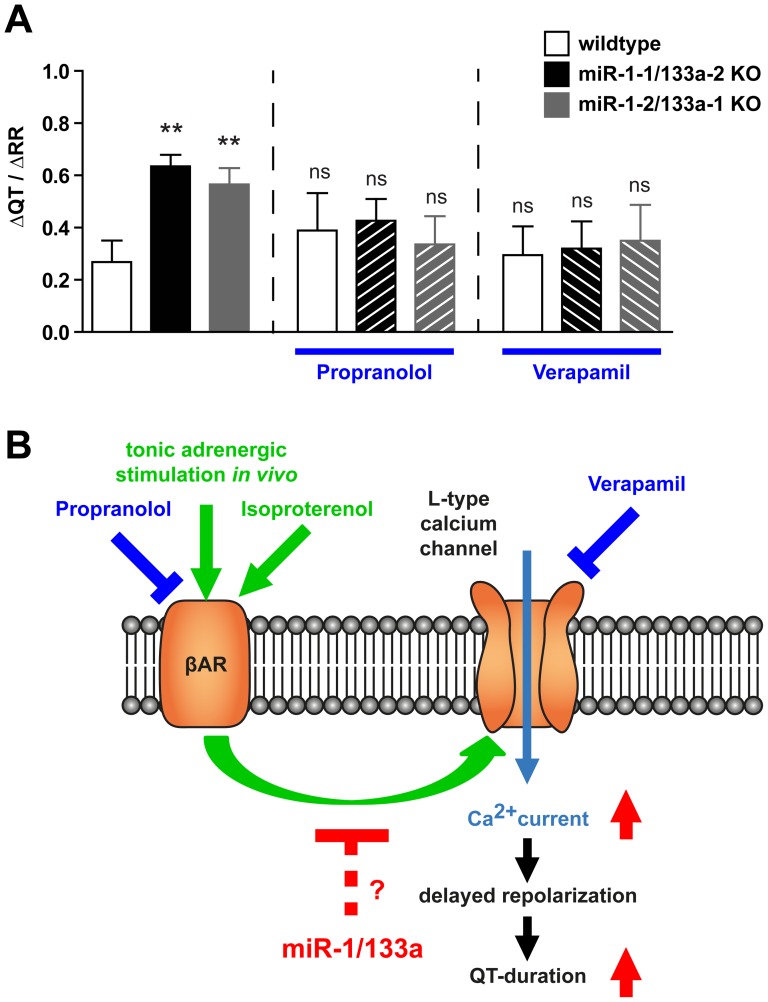
miR-1/133a controls impact of β-adrenergic regulation on L-type calcium-channel. The increased slope of ΔQT/ΔRR indicates LQT at low heart rates in miR-1-1/133a-2 and miR-1-2/133a-1 mutant mice. The LQT was rescued in vivo by inhibition of β-adrenergic signaling using Propranolol or by inhibition of L-type calcium channel using Verapamil, respectively (A). This result confirms the in vitro measurements proving that the miR-1/133a clusters modulate β-adrenergic signaling mediated regulation of L-type calcium channel activity and that loss of this modulation causes LQTS after deletion of single miR-1/133a clusters. Thus the miR-1/133a clusters are essential for repression of the smooth muscle gene program in post-natal heart and for maintenance of repolarization properties that are essential for normal function of the heart in its physiological context (B).

*In vivo* heart function is constantly modulated by the sympathetic and parasympathetic stimuli, thus cardiomyocytes are always subject to regulation by β-adrenergic signaling although to a varying degree. To demonstrate that the dysregulation of adrenergic signaling on calcium-channel activity that was observed *in vitro* is also the cause of the longQT observed *in vivo* in miR-1/133a KO mice, we investigated the effect of adrenergic signaling on ECG in WT and the respective miR-1/133a single cluster *knock-out* mice. Indeed, application of Propranolol and thus blockage of adrenergic signaling *in vivo* abrogated the longQT phenotype in miR-1/133a single cluster *knock-out* mice (ΔQT/ΔRR, p-values compared to untreated wildtype, wildtype 0.39±0.14 p>0.44, miR-1-1/133a-2 KO 0.43±0.08 p>0.32, miR-1-2/133a-1 KO 0.34±0.11 p>0.62, n = 7/4/9, Figure 9A). In addition, direct targeting of L-type calcium-channels by Verapamil also abolished the QT duration differences between WT and the respective *knock-out* models (ΔQT/ΔRR, p-values compared to untreated wildtype, wildtype 0.30±0.11 p>0.85, miR-1-1/133a-2 KO 0.32±0.10 p>0.75, miR-1-2/133a-1 KO 0.35±0.14 p>0.62 n = 6/4/4; Figure 9A). Altogether these data support the notion that dysregulation of L-type calcium channel activity by disturbed impact of ß-adrenergic signaling is the cause of LQT in mice with reduced expression of miR-1/133a (Figure 9B).

## Discussion

Our analysis of single miR-1/133a clusters in a mixed 129/C57 genetic background indicated that the single miR-1/133a clusters are not essential for early development and structural integrity of the heart. However, reduction of miR-1/133a abundance in the single cluster *knock-out* mice revealed other important physiological functions of these miRNAs. Our thorough analysis of ECG parameters uncovered that the miR-1/133a clusters are needed to maintain specific functions in heart electrophysiology and modulation of miR-1/133a dosage caused a striking change in the frequency dependent modulation of QT duration leading to long QT disease phenotype that becomes particularly evident at lower heart rates.

### LQT in miR-1/133a mutant mice is caused by unleashed β-adrenergic control of L-type calcium channel

QT duration is primarily regulated by the function of ion channels and these ion channels are modulated by changes in the activity of interacting molecules and it becomes increasingly clear that the interaction of many components finally determines the electrophysiological properties of the heart [29]. This tight regulation of QT-duration is essential for general heart function to prevent potentially fatal arrhythmias and on the other hand these properties have to be fine-tuned to the functional requirements of a species. Deletion of a single miR-1/133a cluster led to longQT and this was corroborated by significantly prolonged APD_90_ duration at the single cell level. However, our detailed analysis of the electrophysiological properties of isolated cardiomyocytes from both miR-1/133a *knock-out* models did not reveal striking alterations of potassium and sodium current expression and function. Instead, we detected an increased L-type calcium channel activity. This became visible when we mimicked the tonic adrenergic stimulation of the cardiomyocytes that naturally occurs in the intact heart. Increased L-type calcium channel activity has been identified to be responsible for the LQT8 observed in human Timothy's syndrome and the influence of β-adrenergic signaling on this ion-channel and on longQT is well known. Enhanced L-type calcium channel activity could lead to prolonged APD either by longer and stronger depolarization during the plateau phase of APs or by enhanced Ca^2+^ flux though the channel. The larger amount of Ca^2+^ subsequently needs to be exported by the electrogenic Na^+^-Ca^2+^ exchanger which leads to inward currents and delayed (after-) depolarization upon Ca^2+^ removal. Thus also altered intracellular Ca^2+^ buffering in KO cardiomyocytes could enhance the effects of enhanced I_Ca,L_ on the Na^+^-Ca^2+^ exchanger and APD prolongation. In contrast to loss-of-function mutation in LQT1 patients which leads to prolonged QT durations and induction of ventricular tachycardia at higher heart rates [30] in the miR-1/133a mutant mouse models we have detected the opposite effect on QT/RR slope, namely prolongation of action potential duration at lower heart rates. This is very similar to the situation reported in LQT3 patients [31] and mice [24] due to a gain of function mutation of the voltage dependent Na^+^ channel, that shows the most prominent effect at slow heart rates. We believe that the miR-1/133a KO - induced gain of function effects on the Ca^2+^ currents after β-adrenergic stimulation is similarly leading to prolonged QT durations at slow heart rate. This is supported by the fact that both the β-blocker Propranolol as well as the Ca^2+^ channel blocker verapamil attenuates the miR-1/133a KO - induced effects on the QT/RR slope *in vivo* (Figure 9A).

Although Cacna1c, coding for Cav1.2 protein, which is the α-subunit of the L-type calcium channel was suggested to be a miR-1 target in humans [17], we could not confirm direct regulation of Cav1.2 protein abundance by miR-1 in mouse cardiomyocytes, obviously because the miR-1 binding sites are not sufficiently conserved in mouse Cacna1c. We even observed a decrease in protein abundance for the Cav1.2 α-subunit of the L-type calcium channel in our models, arguing for secondary effects but not for release from miRNA mediated repression in the mutant animals.

The activity of the L-type calcium channel is modulated by modification and interacting molecules [32], [33]. We therefore assumed that other, probably ß-adrenergic signaling dependent mechanisms might cause an increased L-type calcium channel current in isolated cardiomyocytes. To understand the molecular mechanisms, how miR-1/133a might cause the changed L-type calcium channel activity, we analyzed adrenergic signaling in cardiomyocytes isolated from WT and the respective miR-1/133a *knock-out* animals. Although several components of the adrenergic signaling cascade are potential direct targets of repression by miR-1 or miR-133a no particularly significant change in the abundance of these molecules *in vivo* or in isolated cardiomyocytes was detected. This was different compared to a recent study using transgenic overexpression of miR-133 [34], but in line with our data that showed that there is no hypertrophic growth after loss of single miR-1/133a clusters [7] that could potentially be attributed to increased adrenergic signaling [28]. Thus we functionally analyzed the adrenergic signaling cascade further downstream by determination of the phosphorylation status of several of its components with and without adrenergic stimulation. For several of the known components of this signaling we did not detect differences in phosphorylation at baseline or after adrenergic stimulation compared to WT controls. Of note, no changes in well-known targets of PKA mediated β-adrenergic signaling were observed. Interestingly, there is a significant change in the CaMKII dependent RyR2 Ser2814 phosphorylation at baseline and after stimulation confirming that miR-1/133a directly or indirectly affects β-adrenergic signaling of miR-1/133a mutant cardiomyocytes. RyR2 Ser2814 has been identified to be regulated by PP2A regulatory subunit B56α that is a candidate miR-1 target gene [8], [9]. However, the B56α subunit was not regulated in cardiomyocytes with reduced miR-1/133a expression. Moreover, reduced CaMKII signaling should reduce activity of the L-type calcium channel [35], thus this change cannot be accounted for the increased L-type calcium channel activity seen in the miR-1/133a single cluster mutant cells. Our unbiased analysis of transcriptional changes after mutation of single miR-1/133a clusters revealed the miR-1 target Sorcin [36] to be significantly upregulated at least in miR-1-1/133a-2 *knock-out* mice and we could confirm this finding at the protein level. Whereas it is established that Sorcin may enhance contractility of cardiomyocytes, it is currently unclear whether Sorcin might also modulate L-type calcium channel activity *in vivo* [37]. We found moderate upregulation of Sorcin only in one of the models. Thus we exclude Sorcin to be the cause of longQT in both models. Taken together, although we did not identify a single miR-1 or miR-133a target molecule responsible for the modulation of adrenergic signaling in our models, numerous molecules related to this pathway are potential targets of miR-1/133a regulation. We suggest that the sum of subtle changes governed by miR-1/133a regulates the impact of adrenergic signaling on L-type calcium channel activity and thus caused LQT in the models with reduced miR-1/133a expression.

### Mutation of single miR-1/133a clusters reveal molecular targets modulated by physiological levels of miRNA regulation in cardiomyocytes

The deletion of the single miR-1/133a clusters offers the opportunity to modulate the miR-1/133a dosage in a way that closely matches the physiological relevant regulation. *In vivo* transcriptional regulation of miR-1/133a clusters would affect both miRNAs within the cluster, and modulation of miRNA abundance without complete loss of expression occurs also in pathophysiological settings [38].

Reduction of miR-1 to approximately 70% (miR-1-2/133a mutants) or 40% (miR-1-1/133a-2 mutants) and concomitant miR-133a reduction revealed gradual regulation of several previously described miR-1/133a targets like Kcne1 [18] and connexin43 [39] and SRF [6]. However, the observed regulation of Kcne1 and connexin43 did not have obvious functional consequences. Nevertheless, the regulatory interaction between miR-1 and Kcne1 might be of more importance in humans and indicates that KCNE1 might be added to a miR-1/133a regulated network of genes that modulates repolarization of cardiomyocytes. Increased abundance of IRX5 protein after loss of miR-1-2 [2] and of IRX5 mRNA after loss of miR-1-1 [5] has been reported. Our analysis revealed that Irx5 protein expression was not increased in the hearts of the mouse mutants described here. In accordance with this, we also did not see regulation of the putative IRX5 downstream gene Kcnd2 that has been observed in the miR-1-2 mutants, moreover we did not observe the QRS complex abnormalities or arrhythmogenesis that were at least in part attributed to the dysregulation of a IRX5-Kcnd2 axis [2].

### miR-1/133a controls expression of smooth muscle genes in cardiomyocytes

Deletion of miR-1-1/133a-2 revealed a moderate but significant increase of SRF protein in mutant cardiomyocytes, whereas this increase was not significant in miR-1-2/133a-1 mutant cardiomyocytes. The miR-1 target myocardin, that is a SRF interacting transcriptional coactivator, was found to be increased after deletion of both miR-1/133a clusters in embryonic heart [7] or after complete deletion of both miR-1 copies in neonatal heart [5], but was not significantly upregulated in the adult single cluster mutant cardiomyocytes. These results demonstrate that the regulatory power of miRNA strongly depend on the actual changes in miRNA abundance.

Although the miR-133a target SRF was increased in miR-133a double mutant hearts [6], it was not increased in embryonic hearts after complete deletion of miR-1/133a [7], indicating that there are also developmental stage specific components modulating miRNA mediated regulation of target protein abundance. Hereby, our analysis supports the view that only careful consideration of experimental miRNA levels in the specific physiological background is useful to reveal physiological functions of miRNAs. Indeed only after more than 50% reduction of mature miR-1 and miR-133a we observed a moderate impact on SRF expression and concomitant increase of few smooth muscle markers in the adult heart. However, even in the more severely affected miR-1-1/133a-2 model we observed no considerable impact on functional parameters of the heart under physiological conditions.

In summary, we suggest that modulation of components of adrenergic signaling regulating the action potential duration of cardiomyocytes might be subject to complex regulation by miR-1 and miR-133a. In line with the common models of miRNA action the longQT phenotype after loss of a single miR-1/133a cluster seems not to be caused by dysregulation of a single ion-channel or a single ion-channel interacting protein, thus we suggest that the miR-1/133a cluster fine-tune different components of the adrenergic signaling to the needs of physiological function *in vivo*. Indeed the rescue of the long QT-phenotype either by inhibition of the adrenergic signaling or by Verapamil-induced modulation of L-type calcium channel activity *in vivo* indicates that the miRNAs miR-1 and miR-133a modulate an important functional property of the heart (Figure 9). In other vertebrates this impact might be developed differently. Kcne1 for instance has a prominent role in repolarization in humans or Cav1.2 as a part of L-type calcium-channel is regulated by miR-1 due to functional miR-1 binding sites in humans. Therefore we suggest that miR-1/133a clusters participate in fine-tuning the regulation of cardiac repolarization according to the needs of the different species. A more profound understanding of the miRNA-mediated mechanisms regulating QT duration may help to develop appropriate therapeutic strategies to prevent the potentially fatal arrhythmias.

## Supporting Information

Figure S1 **Deletion of the intronic miR-1-2/133a-1 cluster does not affect the expression of the host gene Mib1.** RT-PCR using oligonucleotides directed against exons flanking the miR-1-2/133a-1 containing intron (A) indicates that splicing of the Mib1 gene is not disturbed (B) despite deletion of the miR-1-2/133a-1 encoding region of the Mib1 intron. (C) qRT-PCR indicates that also the abundance of Mib1 mRNA is unchanged in the miR-1-2/133a-1 knock-out mice described here.
(TIF)

Figure S2 **Loss of miR-1/133a single clusters does not impair muscle structure.** Histological analysis of muscle structure did not reveal changes in TA muscle of single cluster mutant mice compared to WT. (A) Fiber size distribution and (B) number of centralized nuclei was not changed. (C, D) Type 1 fiber staining indicates no change in fiber type distribution. Comparable regions of TA muscle stained for slow myosin (Sigma) are depicted in D. The scale bar corresponds to 100 µm.
(TIF)

Figure S3 **Macroscopic IKs is not detected in adult control and miR-1-2/133a-1 KO ventricular cardiomyocytes, but in control embryonic cardiomyocytes.** Representative voltage clamp recordings in wildtype embryonic (E14.5–16.5) (A, B) and in control (C) and miR-1-2/133a-1 knock-out (D) adult cardiomyocytes to detect IKs: the three different voltage recordings were performed in presence of a selective blocker of IKr (1 µM E4031; 1 black), of Isoproterenol (1 µM ISO; 2 red), and of Isoproterenol and a selective IKs blocker (1 µM Chromanol; 3 blue). Note the slowly activating outward current in the embryonic cardiomyocyte after Isoproterenol application, which could be blocked by Chromanol indicative for IKs, whereas this IK component could not be detected in control and miR-1-2/133a-1 KO adult ventricular cardiomyocytes. The time course of peak IK of the cardiomyocytes shown in B is displayed in A, IKs was elicited by 5 s long depolarizing voltage steps to +50 mV, followed by a step to 0 mV, holding potential −40 mV, rate 0.05 Hz.
(TIF)

Figure S4 **The IK blocker 4-Aminopyridine (4-AP) has similar effects in control and miR-1-1/133a-2 KO ventricular cardiomyocytes.** AP recordings in respective miR control (left) and KO cells in normal solution (NS, black and blue traces) and after application of 4-AP (2 mM, red traces). (B) APD90 for miR-1-1/133a-2 (left panel) and miR-1-2/133a-1 control and KO cells (right panel). (C) % of increase of the APD90 in control and KO cells upon application of 4-AP (APD90 prolongation in presence of 4-AP for miR-1-1/133a-2 control cells 208.5±31.6, n = 14, for miR-1-1/133a-2 KO cells, 158.3±30.5, n = 13; for miR-1-2/133a-1 control cells 80.9±10.1%, n = 10, for miR-1-2/133a-1 KO cells, 54.1±11.1%, n = 14).
(TIF)

Figure S5 **INa is similar in miR-1-2/133a-1 control and KO ventricular cardiomyocytes.** Representative INa traces recorded from miR-1-2/133a-1 control (A, left) and KO (A, right) ventricular cardiomyocytes in response to 40 ms lasting depolarizing pulses from −80 mV to −10 mV in 10 mV intervals, holding potential −100 mV. The depicted traces were recorded at −10 mV. (B) Statistics of peak INa density at the step potential of −10 mV in both groups of cells. (C) Representative analysis of recovery from inactivation of peak INa measured at 2 mM extracellular Na+; INa amplitude was normalized with the first voltage step to −10 mV, holding potential −100 mV. (D) Statistical analysis of the exponential fit of the recovery from inactivation kinetics of INa.
(TIF)

Figure S6 **Molecules affecting L-type calcium channel activity.** Western blot analysis reveals unchanged expression of the potential miR-1 targets B56α (A) and a significant increase in protein abundance of Sorcin (SRI) in miR-1-1/133a-2 mutant hearts (B). The abundance of the L-type calcium channel beta subunit CAVB2 is not changed (C).
(TIF)
