# Supplementary figures and images for: MiRNA-1/133a Clusters Regulate Adrenergic Control of Cardiac Repolarization
Source: PLoS One. 2014 Nov 21;9(11):e113449. doi: 10.1371/journal.pone.0113449 (PMC4240597; doi:10.1371/journal.pone.0113449)

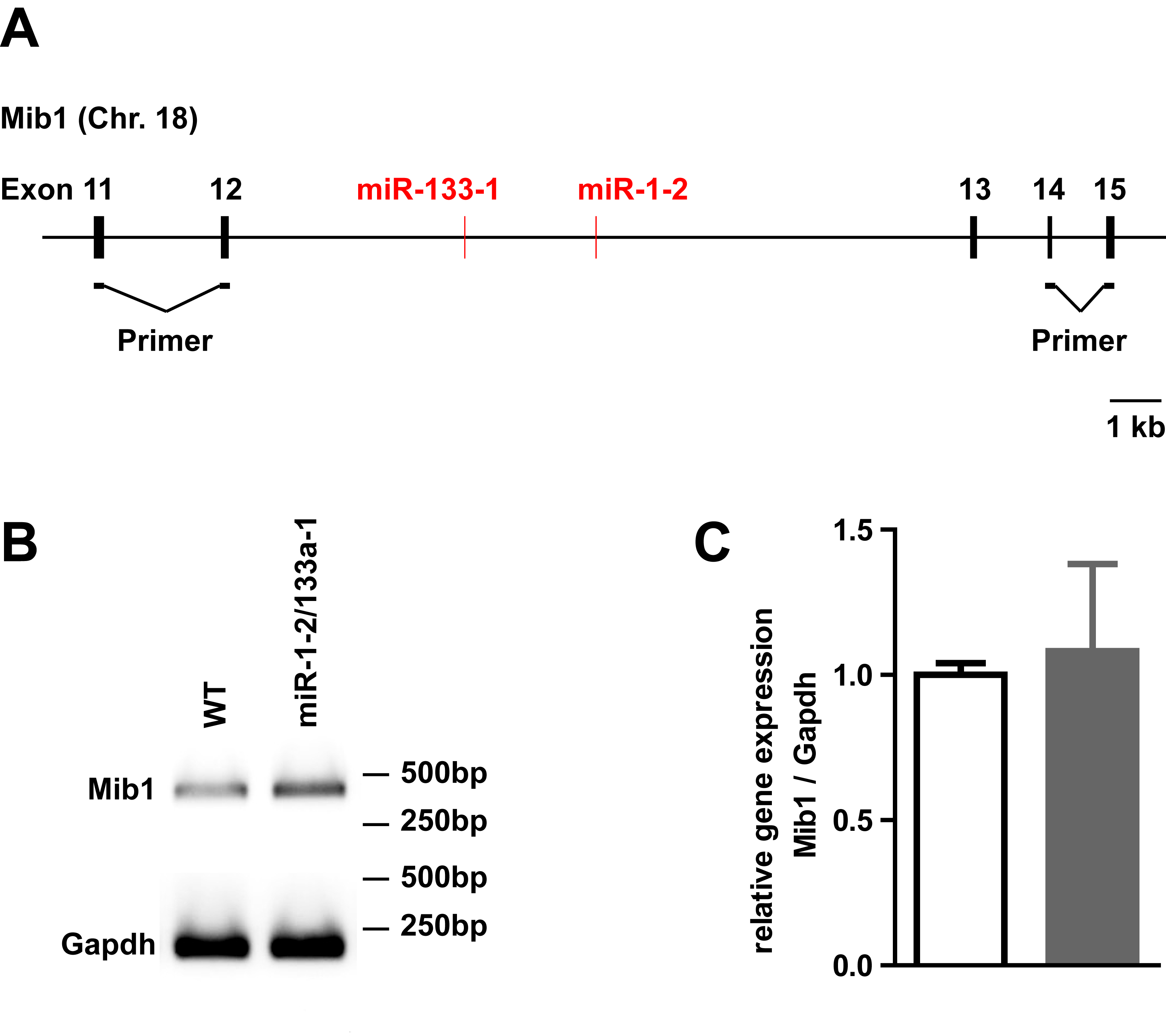

Supplement: Figure S1 — Deletion of the intronic miR-1-2/133a-1 cluster does not affect the expression of the host gene Mib1. RT-PCR using oligonucleotides directed against exons flanking the miR-1-2/133a-1 containing intron (A) indicates that splicing of the Mib1 gene is not disturbed (B) despite deletion of the miR-1-2/133a-1 encoding region of the Mib1 intron. (C) qRT-PCR indicates that also the abundance of Mib1 mRNA is unchanged in the miR-1-2/133a-1 knock-out mice described here. (TIF) [file pone.0113449.s001.tif]

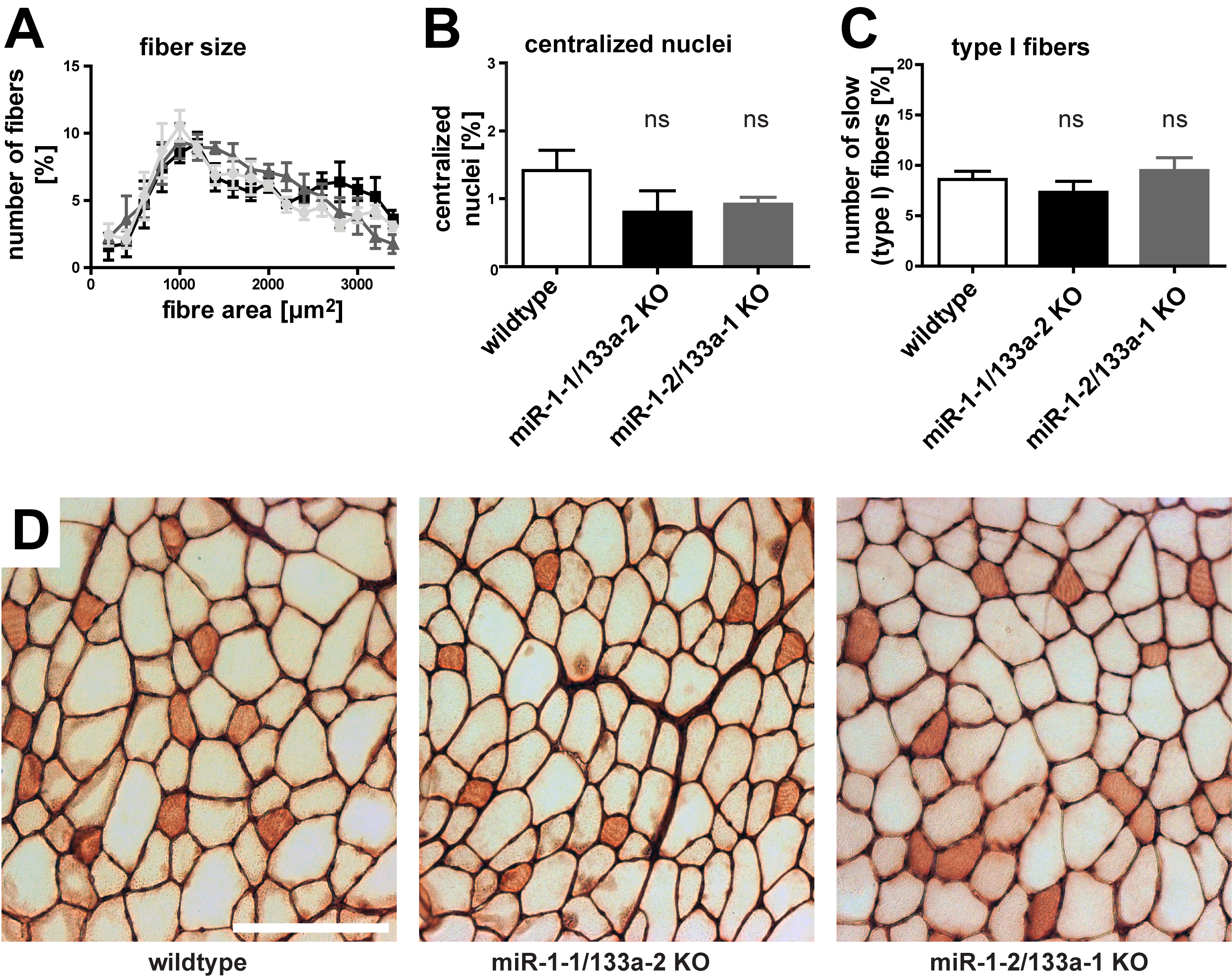

Supplement: Figure S2 — Loss of miR-1/133a single clusters does not impair muscle structure. Histological analysis of muscle structure did not reveal changes in TA muscle of single cluster mutant mice compared to WT. (A) Fiber size distribution and (B) number of centralized nuclei was not changed. (C, D) Type 1 fiber staining indicates no change in fiber type distribution. Comparable regions of TA muscle stained for slow myosin (Sigma) are depicted in D. The scale bar corresponds to 100 µm. (TIF) [file pone.0113449.s002.tif]

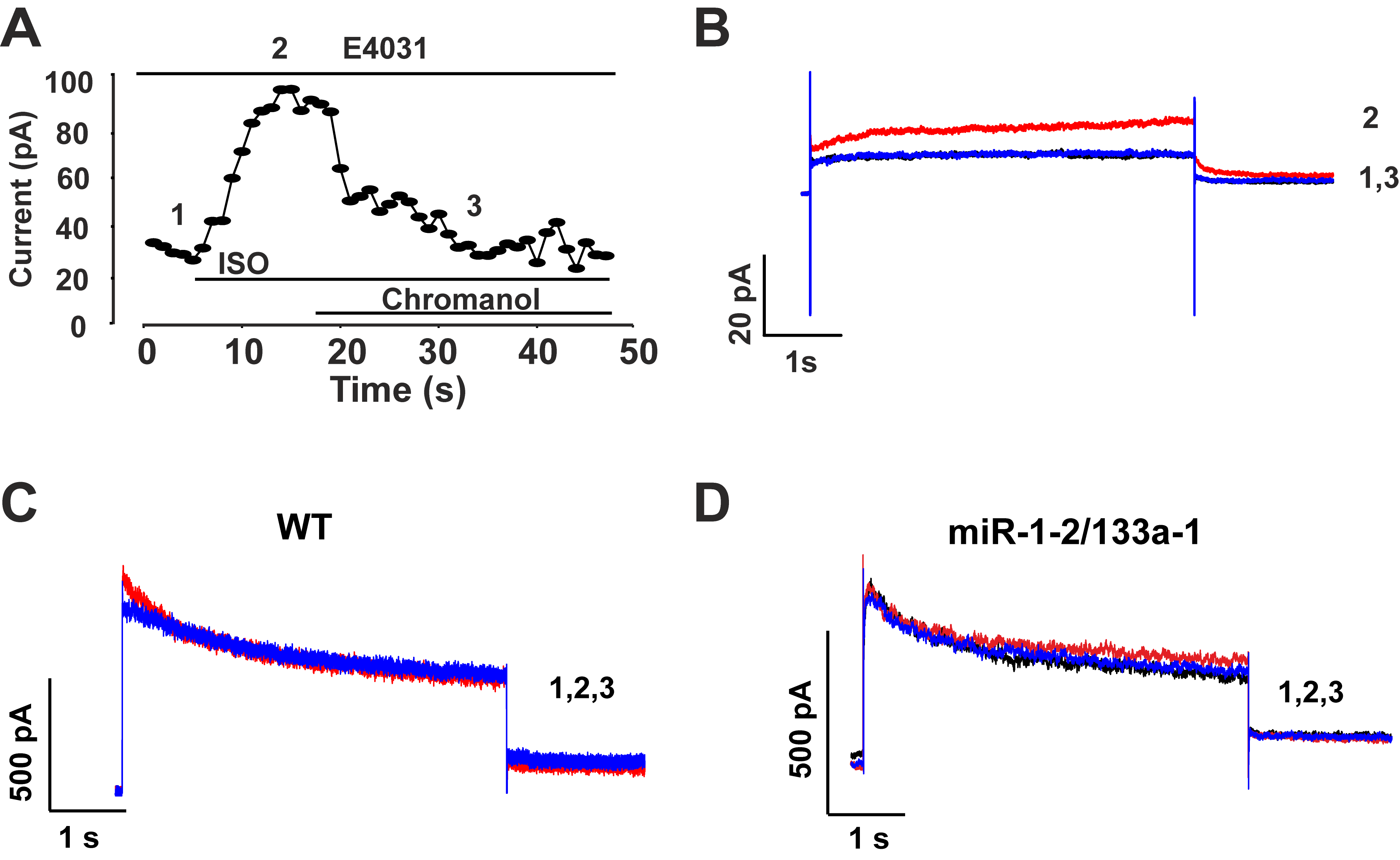

Supplement: Figure S3 — Macroscopic IKs is not detected in adult control and miR-1-2/133a-1 KO ventricular cardiomyocytes, but in control embryonic cardiomyocytes. Representative voltage clamp recordings in wildtype embryonic (E14.5–16.5) (A, B) and in control (C) and miR-1-2/133a-1 knock-out (D) adult cardiomyocytes to detect IKs: the three different voltage recordings were performed in presence of a selective blocker of IKr (1 µM E4031; 1 black), of Isoproterenol (1 µM ISO; 2 red), and of Isoproterenol and a selective IKs blocker (1 µM Chromanol; 3 blue). Note the slowly activating outward current in the embryonic cardiomyocyte after Isoproterenol application, which could be blocked by Chromanol indicative for IKs, whereas this IK component could not be detected in control and miR-1-2/133a-1 KO adult ventricular cardiomyocytes. The time course of peak IK of the cardiomyocytes shown in B is displayed in A, IKs was elicited by 5 s long depolarizing voltage steps to +50 mV, followed by a step to 0 mV, holding potential −40 mV, rate 0.05 Hz. (TIF) [file pone.0113449.s003.tif]

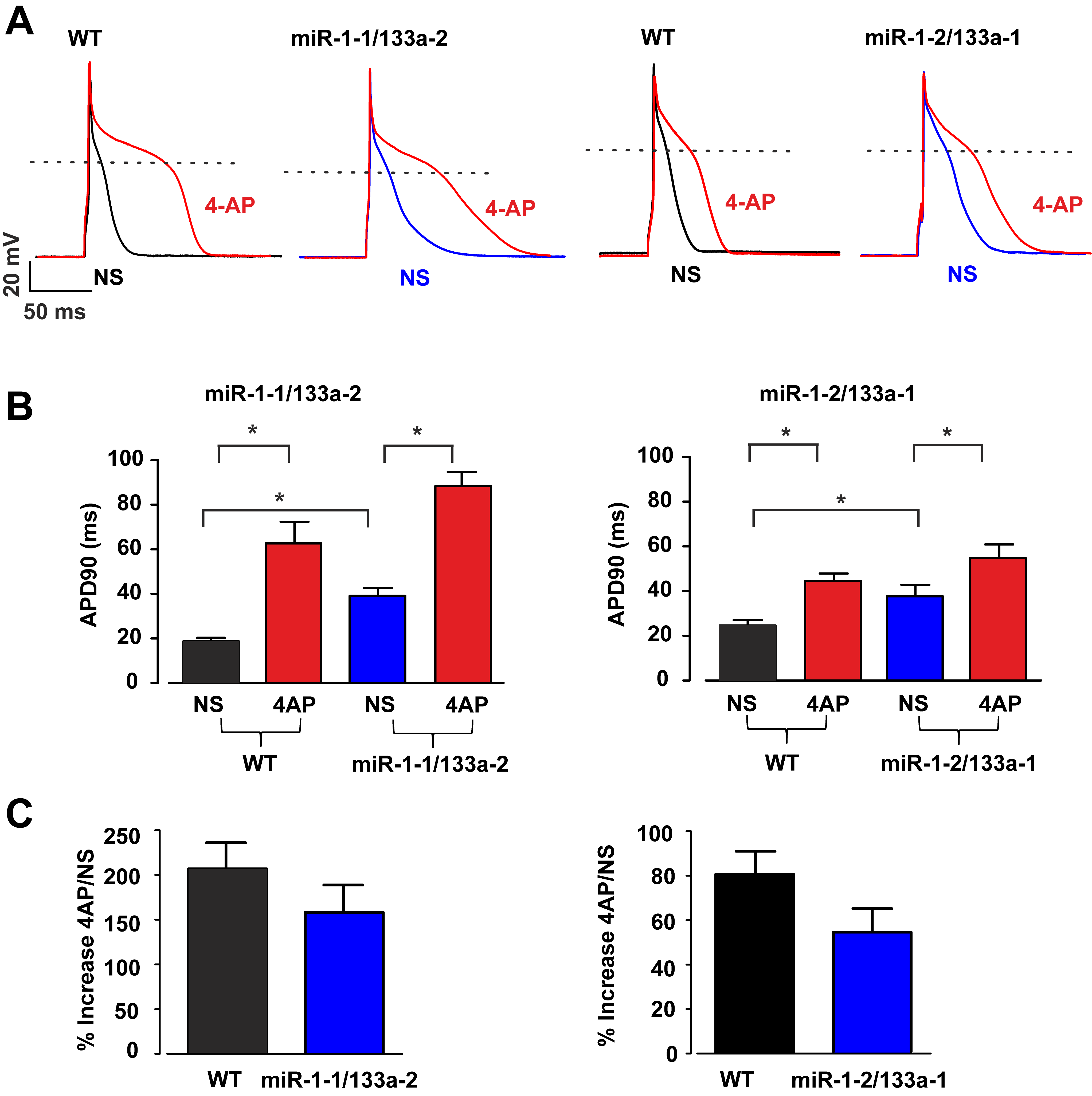

Supplement: Figure S4 — The IK blocker 4-Aminopyridine (4-AP) has similar effects in control and miR-1-1/133a-2 KO ventricular cardiomyocytes. AP recordings in respective miR control (left) and KO cells in normal solution (NS, black and blue traces) and after application of 4-AP (2 mM, red traces). (B) APD90 for miR-1-1/133a-2 (left panel) and miR-1-2/133a-1 control and KO cells (right panel). (C) % of increase of the APD90 in control and KO cells upon application of 4-AP (APD90 prolongation in presence of 4-AP for miR-1-1/133a-2 control cells 208.5±31.6, n = 14, for miR-1-1/133a-2 KO cells, 158.3±30.5, n = 13; for miR-1-2/133a-1 control cells 80.9±10.1%, n = 10, for miR-1-2/133a-1 KO cells, 54.1±11.1%, n = 14). (TIF) [file pone.0113449.s004.tif]

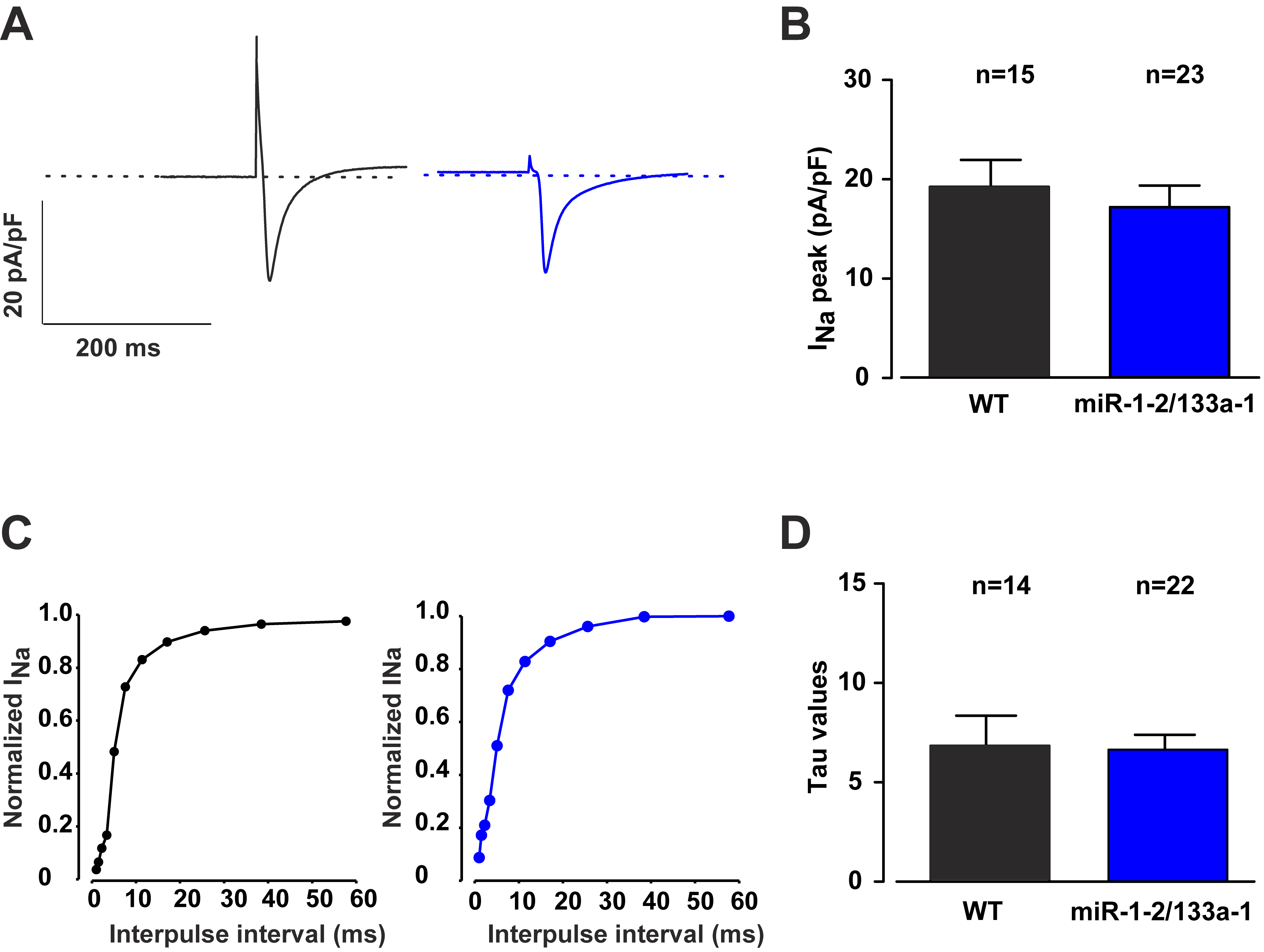

Supplement: Figure S5 — INa is similar in miR-1-2/133a-1 control and KO ventricular cardiomyocytes. Representative INa traces recorded from miR-1-2/133a-1 control (A, left) and KO (A, right) ventricular cardiomyocytes in response to 40 ms lasting depolarizing pulses from −80 mV to −10 mV in 10 mV intervals, holding potential −100 mV. The depicted traces were recorded at −10 mV. (B) Statistics of peak INa density at the step potential of −10 mV in both groups of cells. (C) Representative analysis of recovery from inactivation of peak INa measured at 2 mM extracellular Na+; INa amplitude was normalized with the first voltage step to −10 mV, holding potential −100 mV. (D) Statistical analysis of the exponential fit of the recovery from inactivation kinetics of INa. (TIF) [file pone.0113449.s005.tif]

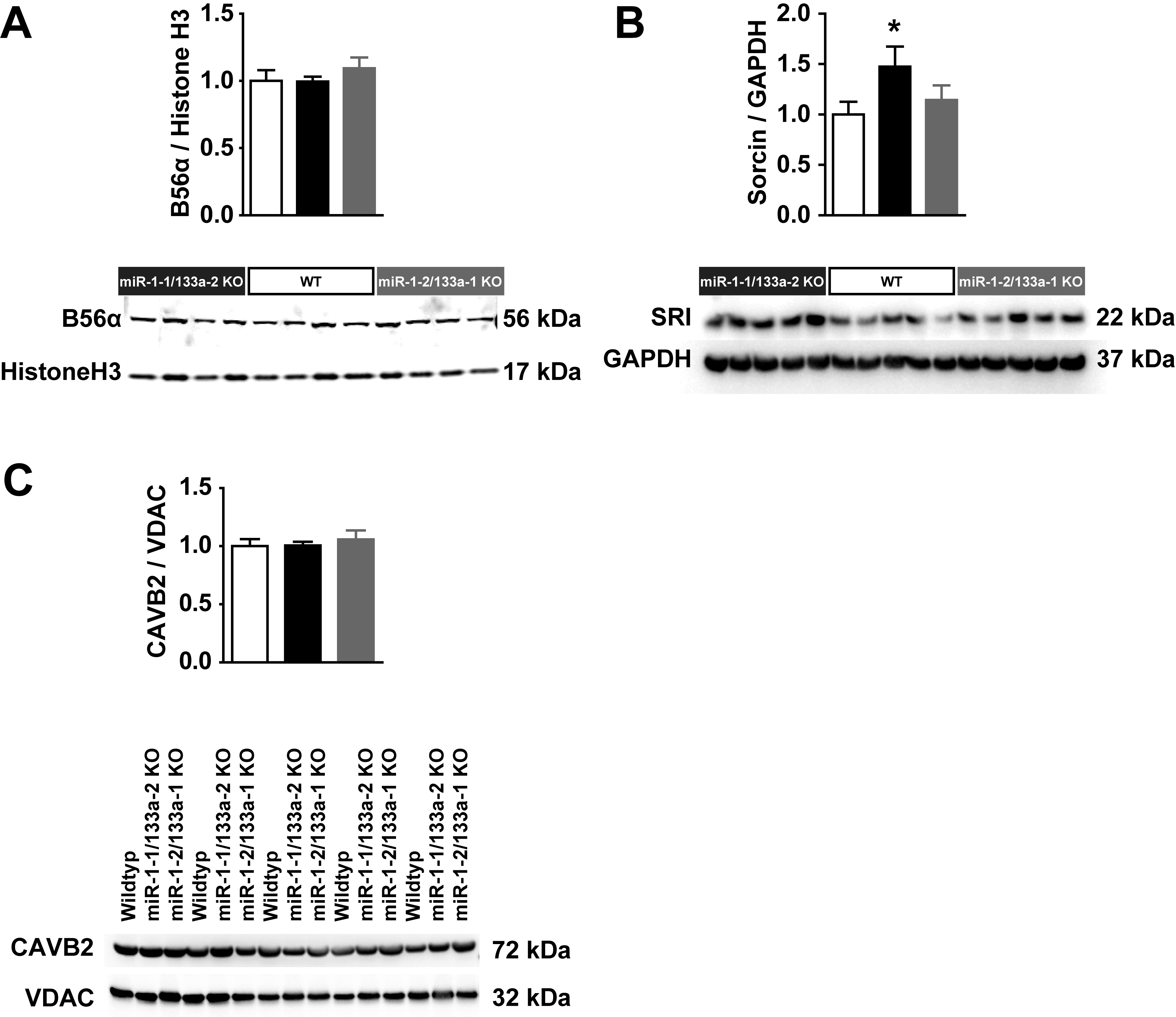

Supplement: Figure S6 — Molecules affecting L-type calcium channel activity. Western blot analysis reveals unchanged expression of the potential miR-1 targets B56α (A) and a significant increase in protein abundance of Sorcin (SRI) in miR-1-1/133a-2 mutant hearts (B). The abundance of the L-type calcium channel beta subunit CAVB2 is not changed (C). (TIF) [file pone.0113449.s006.tif]
